# Anpassung von Cochleaimplantatsystemen

**DOI:** 10.1007/s00106-025-01593-5

**Published:** 2025-04-09

**Authors:** Uwe Baumann, Tobias Weißgerber, Ulrich Hoppe

**Affiliations:** 1 Universitätsmedizin Frankfurt, Schwerpunkt Audiologische Akustik, Klinik für HNO-Heilkunde, Goethe-Universität Frankfurt, Theodor-Stern-Kai 7, 60590 Frankfurt am Main, Deutschland; 2 Audiologische Abteilung, Hals‑, Nasen‑, Ohrenklinik, Kopf- und Halschirurgie, Universitätsklinikum Erlangen, Waldstraße 1, 91054 Erlangen, Deutschland

**Keywords:** Cochleaimplantate, Implantierbare Neurostimulatoren, Prothesen und Implantate, Verifikation, Rehabilitation, Cochlear implants, Implantable neurostimulators, Prostheses and implants, Verification, Rehabilitation

## Abstract

Die technische Anpassung von Cochleaimplantat(CI)-Systemen ist ein zentraler Bestandteil des Versorgungsprozesses. Einerseits besteht dieser aus einem standardisierten Vorgehen, andererseits werden individuelle Bedürfnisse des Patienten berücksichtigt. Sie beginnt mit der „Erstanpassung“ in der Basistherapie, wobei zunächst ein zufriedenstellender Klang- und Lautheitseindruck im Fokus stehen. In der Folgetherapie wird eine Feinanpassung vorgenommen, um ein bestmögliches Sprachverstehen in Ruhe und im Störgeräusch zu erreichen. Ziel ist eine individuelle Anpassung des CI-Prozessors an audiologische Vorgaben. Die Anpassung erfolgt durch ein gemäß der Leitlinie zur CI-Versorgung speziell qualifiziertes Fachpersonal und berücksichtigt anatomische Gegebenheiten, elektrophysiologische Messungen und audiologische Evaluationsdaten. Die CI-Anpassung soll die Hör- und Sprachverständlichkeit lebenslang erhalten und so für eine umfangreiche Verbesserung der Lebensqualität sorgen. Bei Kindern ist die bestmögliche CI-Anpassung Grundlage für eine normale Sprachentwicklung.

## Abstract (en)

The technical fitting of cochlear implant (CI) systems is a central component of the CI care process. On the one hand, this consists of a standardized procedure, while on the other, the individual needs of the patient are taken into account. It starts with the “initial fitting” during basic therapy, where the initial focus is on achieving a satisfactory impression of sound and loudness. In the follow-up therapy, fine adjustment is carried out to achieve the best possible speech perception in quiet and in noise. The aim is to individually adjust the CI processor to audiological targets. The fitting is carried out by a specially qualified professional in accordance with the German CI fitting guidelines and takes into account anatomical conditions, electrophysiological measurements, and audiological evaluation data. The CI fitting should maintain hearing and speech perception throughout life, thus ensuring a significant improvement in quality of life. In children, achieving the best possible CI fitting is fundamental to facilitating normal speech development.

Der Erfolg der Versorgung mit einem Cochleaimplantat (CI) ist maßgeblich von der Anpassung des Systems an die individuellen Bedürfnisse der Patientinnen und Patienten abhängig. Die individuellen Voraussetzungen, Hörerfahrungen und Bedürfnisse CI-versorgter Patientinnen und Patienten variieren erheblich. Auch Personen, die über keine Hörfähigkeit oder nur über eine Resthörigkeit verfügen, können von einer Versorgung mit CI profitieren. Bei Kindern, die von Geburt an taub sind, kann eine Versorgung mit CI in aller Regel bereits vor dem ersten Geburtstag mit Erfolg durchgeführt werden. Die bilaterale Implantation stellt mittlerweile einen etablierten Behandlungsstandard dar, sofern eine Indikation hierfür gegeben ist.

Die Anpassung des zum CI-System gehörenden CI-Prozessors stellt einen Bestandteil der audiologischen Leistungen nach der CI-Indikation dar [2] und erfolgt unter Zuhilfenahme produktspezifischer, durch den Hersteller bereitgestellter Anpassungsprogramme. Die Anpassung und der Betrieb von CI-Systemen unterliegen speziellen Auflagen und Vorschriften des Medizinproduktegesetzes (MPG, [10]) sowie der Medizinproduktebetreiberverordnung (MPBetrV, [11]), welche beide durch das Bundesministerium für Gesundheit erlassen und in der Leitlinie zur CI-Versorgung berücksichtigt wurden [2].

Die angemessene Vorgehensweise bei der Anpassung von CI-Systemen an die individuellen Bedürfnisse der Patientinnen und Patienten erfordert ein breites und vielseitiges Wissen über die entsprechenden Methoden. Nach den Vorgaben der Leitlinie zur CI-Versorgung [2] und des CI-Weißbuchs [47] erfolgt die audiologische Basis- und Folgetherapie sowie die audiologische Nachsorge, die Justierung der Prozessoren einschließt, durch hierfür besonders qualifizierte Audiologen. Die Qualifikationsanforderungen des „CI-spezialisierten Audiologen“ sind im Detail in der jeweils aktuell geltenden CI-Leitlinie der Arbeitsgemeinschaft der Wissenschaftlichen Medizinischen Fachgesellschaften e. V. (AWMF) und dem CI-Weißbuch der Deutschen Gesellschaft für Hals-Nasen-Ohren-Heilkunde, Kopf- und Hals-Chirurgie e. V. (DGHNO-KHC) hinterlegt. Weitere inhaltliche Details zu ergänzenden Qualifikationswegen finden sich auch in Beiträgen der Deutschen Gesellschaft für Audiologie, z. B. „CI-Audiologe (DGA)“ [35], „Audiologischer CI-Assistent (DGA)“ [14].

Im Folgenden werden die einzelnen Schritte der CI-Anpassung vorgestellt. Es werden die Besonderheiten der elektrischen Stimulation und die Konsequenzen für die Anpassung erläutert. Dabei werden sowohl die strukturierten und standardisierten Vorgehensweisen als auch die individuell erforderlichen Modifikationen erläutert.

## Grundlagen der elektrischen Stimulation

Die elektrische Stimulation neuraler Strukturen innerhalb der Cochlea ist ein komplexer Prozess. Es bestehen vielfältige Zusammenhänge zwischen der Anordnung der Stimulations- und Referenzelektrode und der Form des elektrischen Stimulus sowie Abhängigkeiten von der Rate des Pulsmusters und Summationseffekten, die sich aus der räumlich-zeitlichen Verteilung des elektrischen Felds ergeben. Zusätzlich können Beeinträchtigungen der Funktion des Hörnervs (z. B. nach durchlaufener Meningitis), Besonderheiten der Lage des Elektrodenarrays innerhalb der Cochlea (z. B. Skalendislokation der Elektrode) oder Eigenheiten der Leitfähigkeit der knöchernen Struktur des Felsenbeins (z. B. Otospongiose) zu schwer vorherzusagenden Effekten auf die am Ende der Rehabilitation erreichte individuelle Hörleistung mit dem CI-System führen.

### Neuronale Stimulation

Die elektrische Stimulation intracochleärer Elektroden erzeugt elektrische Felder in der Cochlea, die Spannungsgradienten und Stromflüsse in den Hörnerven auslösen. Diese Felder führen durch Depolarisation der Nervenmembranen zur Entstehung von Aktionspotenzialen, die entlang der Axone zum Nucleus cochlearis wandern. Gleichzeitig entstehen neuronale Reaktionen in einer Zellpopulation. Um Gewebeschäden zu vermeiden, werden ladungsausgeglichene Impulse verwendet. Die kathodische Phase eines biphasischen Impulses bewirkt Depolarisation und Spike-Generierung, während die anodische Phase Hyperpolarisation und Ladungsausgleich fördert. Modelle vermuten 2 Spike-Generatoren an zentralen und peripheren Axonen [36].

### Elektrodenkonfiguration

Die Position der indifferenten (Masse‑)Elektrode beeinflusst entscheidend das elektrische Feld bei Cochleaimplantaten (CI). Es gibt 2 Hauptkonfigurationen:

#### Bipolare Anordnung

Die indifferente Elektrode befindet sich in der Cochlea, nahe der aktiven Elektrode. Dies führt zu einer lokalen Feldausbreitung, wodurch ein erheblicher Teil des elektrischen Stroms als Querstrom verloren geht und der Hörnerv weniger effektiv stimuliert wird.

#### Monopolare Anordnung

Eine extracochleäre Elektrode, z. B. am Stimulatorgehäuse oder im Bereich des M. temporalis, dient als Referenz. Diese Konfiguration erzeugt ein breiteres elektrisches Feld ohne Querstromverluste, benötigt jedoch geringere Stimulationspegel (10–20 dB niedriger) und wird klinisch bevorzugt.

#### Besonderheiten und Alternativen

Die „Common-Ground-Verschaltung“ verwendet die übrigen Elektroden des Arrays als Referenz, was bei der Fehlerdiagnose (z. B. Kurzschlüsse) nützlich ist.

Studien untersuchten tri- und quadrupolare Anordnungen, die eine stärkere Feldfokussierung ermöglichen sollen [8, 15, 41].

Die monopolare Konfiguration wird standardmäßig eingesetzt und nur in Sonderfällen, wie bei Fazialis-Kostimulation oder Elektrodendefekten, angepasst.

### Stimulationsstrategie

Mit dem Begriff „Stimulationsstrategie“ wird die Art und Weise der Generierung der elektrischen Pulsmuster zur räumlich-zeitlichen Verteilung der elektrischen Stimulation bezeichnet. Viele Stimulationsstrategien verwenden eine fixe, höherfrequente Träger-Impulsrate, deren Amplitude durch die aus dem akustischen Eingangssignal abgeleitete Hüllkurve moduliert wird. Bei der „channel-specific sampling sequence“ („fine structure processing strategy“, FSP-Strategie) wird im apikalen Bereich des Elektrodenarrays zusätzlich die Reizrate in Abhängigkeit von der Frequenz des jeweiligen Bandpassfilterausgangs instantan angepasst, wovon die Entwickler eine verbesserte Abbildung von Frequenzänderungen erwarten [44]. In Abhängigkeit vom Implantatmodell lassen sich je nach Hersteller teils sehr unterschiedliche Stimulationsstrategien konfigurieren. Im Laufe der Zeit haben sich bewährte Vorgaben für die große Anzahl an Einstellparametern entwickelt, die für die meisten CI-Nutzer einen ausreichenden Hörerfolg gewährleisten. Ein Abweichen von den Herstellerstandards sollte nur dann erwogen werden, wenn ein grundsätzliches Verständnis für die Funktion der Stimulationsstrategie besteht und somit die gewünschte Wirkung der Änderung zu erwarten ist. Eine Darstellung der verschiedenen Stimulationsstrategien findet der interessierte Leser beispielsweise in der Arbeit von Baumann et al. [7].

### Charakteristik der Pulsfolge

Die elektrischen Pulsfolgen von CI können je nach Modell und Hersteller angepasst werden, um die Stimulation zu optimieren. Dabei spielen verschiedene Parameter eine Rolle:

#### Globale Anpassungen

- **Inter-Phase-Gap (IPG):** Die Pause zwischen den beiden Phasen eines bipolaren Pulses kann bei einigen Herstellern variiert werden.
- **Triphasische Pulse:** Geräte des Herstellers MED-EL (Innsbruck, Österreich) können triphasische Pulse erzeugen, um eine unbeabsichtigte Fazialis-Stimulation zu reduzieren [3, 45].
- **Synchronisation:** Die Pulsgenerierung kann an die Verzögerungszeit eines Hörgeräts am Gegenohr angepasst werden, um das Richtungshören zu verbessern (MED-EL) [64].

#### Kanalspezifische Anpassungen

- **Pulsdauer:** Sie ist einstellbar zwischen 10 µs und mehreren 100 µs und beeinflusst maßgeblich die in das Gewebe eingebrachte elektrische Ladung. Bei Amplitudenbeschränkungen durch Erreichen des „Compliance Limits“ wird die Pulsweite vergrößert.
- **Stimulationsrate:** Klinisch übliche Raten pro Kanal liegen zwischen 500 und 2000 Pulsen/s. Niedrigere Raten verschlechtern das Klangbild, höhere Raten steigern den Energiebedarf, ohne das Sprachverstehen zu verbessern [22, 52].

#### Stimulationsstrategien

- **ACE-Stimulationsstrategie (Advanced Combination Encoder):** Eine feste Anzahl der energiereichsten Kanäle (typischerweise 8–12 von bis zu 22) wird ausgewählt, was zu einem klareren Klangbild und einem verbesserten Sprachverstehen bei hoher Energieeffizienz führen soll (Hersteller Cochlear, Macquarie, Australien).
- **Continuous Interleaved Sampling (CIS):** Alle nutzbaren Kanäle werden gleichzeitig stimuliert. Pulsfolgen werden zeitlich versetzt abgegeben, um störende Feldüberlagerungen zu vermeiden (Hersteller Advanced Bionics, Valencia, USA; Cochlear; MED-EL).
- **Mischformen aus CIS und Ratencodierung (FSP, FS4): **ein (FSP) bis 4 (FS4) apikale Kanäle werden mit dem Bandpassfiltersignal entsprechender Rate stimuliert, während basale Elektroden das Signal mit CIS codieren. Eine bessere Auflösung der Tonhöheninformation lässt sich hierdurch erwarten (Hersteller MED-EL).
- **Current Steering:** Simultane Impulse benachbarter Kanäle werden so gewichtet, dass das elektrische Feld zwischen den Kanälen moduliert wird, was die Tonhöhenabbildung verbessern soll (HIRES-Strategien: Hersteller Advanced Bionics, FS4p: Hersteller MED-EL) [4].

Diese vielseitigen Anpassungsmöglichkeiten ermöglichen eine individuell optimierte CI-Anpassung, um Hörqualität und Energieeffizienz zu maximieren. (Übersicht hierzu in der Arbeit von Baumann et al. [7]).

### Dynamikbereich der elektrischen Stimulation

Bei den derzeit klinisch angewendeten CI-Systemen muss für alle verfügbaren Elektroden jeweils eine Unter- und Obergrenze der elektrischen Stimulationspegel für jeden Patienten festgelegt werden (s. Abschnitt „Ablauf der Erstanpassung“). Der zwischen Unter- und Obergrenze liegende Dynamikbereich ist individuell unterschiedlich und liegt im Mittel zwischen 10 und 20 dB. Da die Hersteller bei den für die Einstellung verwendeten Programmen eigene Skalen für die Anzeige des Strompegels eingeführt haben (z. B. „clinical units“, CU), kann nur über Umrechnungen der tatsächliche Stromfluss ermittelt werden. Beispielsweise können Implantate des Herstellers Cochlear (Macquarie, Australien) die Amplitude des Stimulationsstroms in einem in 256 Stufen (CU-Einheiten) unterteilten Bereich variieren. Der CU-Bereich von 0 bis 255 entspricht dabei etwa 10 μA_peak_ bis 1750 μA_peak_. Ein Stromstärkeschritt beträgt hierbei 2,046 % (0,176 dB) der Stromänderung [63].

Bei Vorliegen des „t-Tail-Effekts“ kommt es bei Erhöhung des Strompegels über der Hörschwelle zunächst nicht zu einer für den betroffenen Patienten deutlich bemerkbaren Erhöhung der Lautheitsempfindung. Erst deutlich über der Hörschwelle liegende Stimulationspegel führen in diesen Fällen zu einem regulären Anwachsen der Lautheit. Die t‑Tail-Charakteristik führt zu einem vermeintlich vergrößerten Dynamikbereich und zu einer zu schwachen Übertragung von Signalen geringer Intensität.

## Prozess der CI-Anpassung

Gemäß dem Weißbuch „CI-Versorgung“ ist der Versorgungsprozess in die 3 Phasen „Basistherapie“, „Folgetherapie“ und „Nachsorge“ gegliedert [47], wobei die Anpassung der CI-Systeme Gegenstand aller 3 Phasen ist. Die Bezeichnung „Basistherapie“ bezieht sich auf die initialen Phasen der Aktivierung des CI-Prozessors nach CI-Implantation. In der Regel sind hierzu 3–5 Termine in kurzen Abständen erforderlich. Sofern erforderlich, kann die Basistherapie in mehreren Sitzungen an aufeinanderfolgenden Tagen durchgeführt werden. In der Vergangenheit wurde die erste Aktivierung des CI in der Regel erst nach einer Einheilungsphase von 4–6 Wochen durchgeführt. Der Einsatz einer minimal-invasiven Chirurgie mit stark verkleinerter Schnittführung und hierdurch reduzierter postoperativer Schwellung ermöglicht bei ausreichender Wundheilung eine erste Anpassung auch schon 2–4 Tage nach der Implantation [9, 28]. Dadurch lässt sich der Beginn der sich an die Erstanpassung anschließenden „Folgetherapie“ (CI-Rehabilitation) deutlich früher terminieren, und der Patient kann sehr viel früher erste Hörerfahrungen mit dem CI erhalten.

Die Anwendung interaktiver psychoakustischer Verfahren, welche sich in der Audiologie und der Hörgeräteanpassung bereits bewährt haben, erweist sich als vorteilhaft zur Bestimmung der individuell bestmöglichen Ankopplung der elektrischen Stimulationsmuster an den Hörnerv. In der Anpassung von CI-Systemen bei kleinen Kindern und unkooperativen Patientinnen und Patienten sind objektive Verfahren besonders hilfreich (s. Abschnitt „CI-Anpassung auf der Basis objektiver Messungen“).

### Ablauf der Erstanpassung

**Abb. 1:**
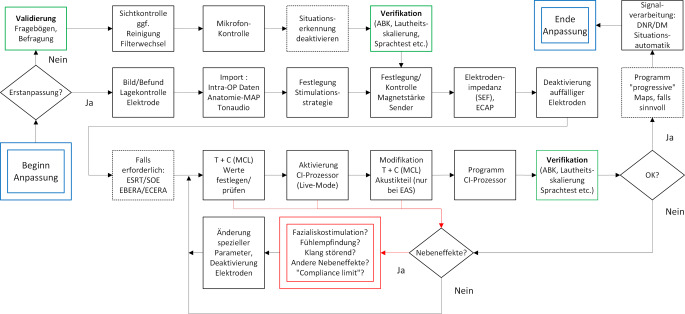
Ablaufschema zur Cochleaimplantat(CI)-Anpassung. *ABK* Aufblähkurve, *C* *(MCL)* „comfortable/most comfortable loudness levels“, *DM* „directional microphone“, *DNR* „digital noise reduction“, *EAS* elektrisch-akustische Stimulation*, EBERA* „electrical brainstem-evoked response audiometry“, *ECAP* „electrically evoked compound action potential“, *ECERA* „electrical cortical-evoked response audiometry“, *ESRT* „electrically evoked stapedius reflex“, *SEF* „spread of electrical field“, *SOE* „spread of exitation“, *T* „threshold“

Die Erstanpassung eines CI ist ein zentraler Schritt im Behandlungsprozess. Dabei wird der externe Prozessor erstmals individuell auf den Nutzer abgestimmt, programmiert und aktiviert. Ein Ablaufschema der Erst- und Folgeanpassungen ist in Abb. 1 dargestellt.

Bei allen Patienten sollte zur Erstanpassung eine radiologische Prüfung der Lage des Elektrodenträgers vorliegen. Unter Berücksichtigung entsprechender anatomischer Kenntnisse gestatten diese Aufnahmen bei unvollständiger Insertion eine Abschätzung der Anzahl der außerhalb der Hörschnecke liegenden Elektrodenkontakte. Im Vergleich zu den bisher üblichen Projektionsradiographien nach Stenvers bieten DVT-Aufnahmen (digitale Volumentomographie) mit Rekonstruktionen zur Darstellung des Elektrodenträgers eine höhere Auflösung.

Im Rahmen der technischen Vorbereitung des CI-Prozessors ist zunächst eine Auswahl und Regulierung der Stärke des am Sender oder im Single-Unit-Prozessor angebrachten Magneten erforderlich. Mitunter kann es bei Patienten mit stärkerem Hautlappen oder gekräuselten Haaren zu einem schwachen Halt der Sendespule kommen, was in Einzelfällen die Anwendung spezieller Sendespulen erfordert. Nach Festlegung der Stärke des Haltemagneten wird eine Prüfung der elektrischen Kopplung mit dem Implantat durch das Anpassungsprogramm durchgeführt. Bei Vorliegen von starken Schwellungen oder eines ausgeprägt dicken Hautlappens kann möglicherweise eine Reduktion der Qualität dieser Kopplung sowie eine Verringerung der für die Stimulation bereitstehenden Energie beobachtet werden.

Vor der Anpassung der Stimulationsparameter erfolgt eine Überprüfung der elektrischen Impedanz der Reizelektroden. Sofern sich aus der Messung Hinweise auf das Vorliegen von Unterbrechungen oder Kurzschlüssen ergeben, werden die entsprechenden Elektroden deaktiviert.

**Abb. 2:**
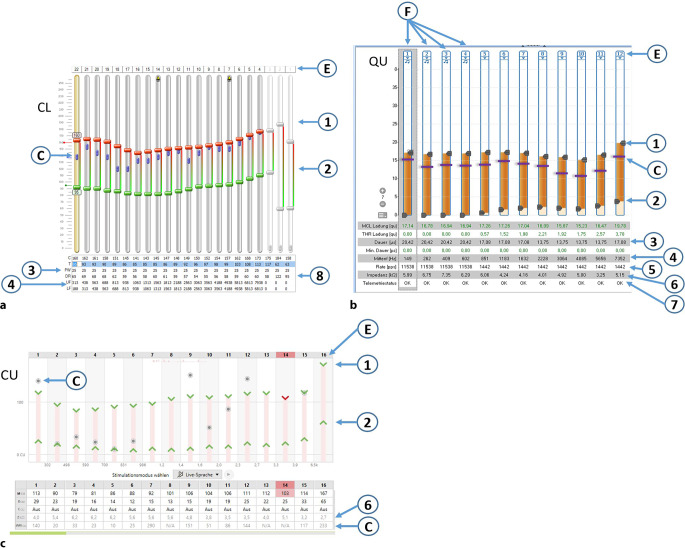
**a** Screenshot des Anpassprogramms Custom Sound CS 7.2 (Fa. Cochlear, Macquarie, Australien). *1 *C‑Werte, *2 *T‑Werte, *3 *Pulsweite, *4 *Bandgrenzen der Filter, *8 *DR – „dynamic range“, *E* Elektroden-Kontaktnummer (E1 basal), *C* eCAP-Schwellen, *CL* „current level“, Einheit der Stimulationsstärke, *eCAP* elektrisch evozierte Summenaktionspotenziale des Hörnervs. **b** Screenshot des Anpassprogramms Maestro 9.x (Fa. MED-EL, Innsbruck, Österreich). *1* MCL-Werte, *2 *THR-Werte, *3 *Pulsweite, *4 *Mittenfrequenz-Filter, *5 *Kanal-Pulsrate, *6 *Elektrodenimpedanz, *7 *Status der Elektrode. *C* eCAP-Schwellen, *E* Elektroden-Kontaktnummer (E1 apikal), *F* Feinstrukturkanäle mit höherer Pulsrate, *QU* „charge units“, Einheit der Stimulationsstärke. **c** Screenshot des Anpassprogramms Target (Fa. Advanced Bionics, Valencia, USA). *1* M-Level, *2* T-Level, *6* Elektrodenimpedanz, *C* eCAP Schwellen. *CU* „clinical units“, Einheit der Stimulationsstärke, *E* Elektroden-Kontaktnummer (E16 basal)

Das Hauptziel der Anpassung ist die frequenz- bzw. elektrodenspezifische Wiederherstellung einer Lautheitswachstumsfunktion, die dem Lautheitsempfinden von Normalhörenden möglichst nahekommt. Im Rahmen der Anpassung erfolgt für jede Elektrode eine Justierung der Amplitude für die Obergrenze und Untergrenze der Stimulation. Außerdem erfolgt ggf. eine Anpassung der Pulsdauer sowie weiterer Parameter. Die Programmieroberflächen der Anpassprogramme der verschiedenen Hersteller sind in Abb. 2a–c dargestellt.

#### Obergrenze der Stimulation

In Abhängigkeit vom Implantattyp ist bei 12–22 Elektroden die Obergrenze des elektrischen Stimulationstroms individuell zu bestimmen. Die Festlegung dieser Obergrenze (Bezeichnung je nach Hersteller C‑, M‑ oder MCL-Wert, „comfortable“ oder „most comfortable level“) erfolgt durch Instruktion des Patienten, die empfundene Intensität nach Präsentation eines elektrischen Reizes anzugeben. In der Praxis findet häufig eine Vorgehensweise Anwendung, die sich an der Methode der kategorialen Lautheitsskalierung orientiert. Dabei wird eine Skala mit einem Intensitätsbereich zwischen „nicht gehört“ und „unangenehm laut“ eingesetzt.

Die Klangqualität des elektrischen Reizes wird von den meisten Patienten als „tonal“ beschrieben. Rauschen oder Verzerrungen des Höreindrucks sind in seltenen Fällen zu beobachten. Der Verlauf des Stromprofils über dem Elektrodenträger kann starken Schwankungen unterliegen, welche durch den individuell variierenden Abstand der Elektrodenkontakte sowie durch die Empfindlichkeit und die Anzahl der noch voll funktionsfähigen Hörnervenfasern verursacht werden. Im Schwellenprofil ist bei basalen Kontakten im Vergleich zu weiter apikal gelegenen Elektroden oberhalb der ersten Cochleawindung häufig ein deutlicher Anstieg zu verzeichnen. Des Weiteren wird der Verlauf durch die Elektrodenkonfiguration beeinflusst. Im Vergleich zur monopolaren Ausbreitung des elektrischen Felds zeigt sich bei bipolaren Verschaltungen ein deutlich unregelmäßigerer Verlauf der gewählten Stimulationspegel.

Sofern an der Obergrenze der elektrischen Stimulation unter Berücksichtigung weiterer Parameter wie der Pulsdauer keine ausreichend hohe Lautheitsempfindung erreicht werden kann, weil störende Nebenempfindungen (beispielsweise Schmerzempfindungen) auftreten, ist eine Deaktivierung der betreffenden Elektroden erforderlich, um eine unerwünschte Stimulation zu vermeiden.

In Anschluss an die initial durchgeführte Bestimmung der Obergrenze der Stimulation wird eine sequenzielle Stimulation aller aktivierbaren Elektrodenkanäle empfohlen, um eine vergleichende Kontrolle der Intensität des Höreindrucks zu ermöglichen. Dabei sollte auch die erwartete Tonotopie kontrolliert werden.

#### Prüfung der Tonotopie

Die durch die elektrische Stimulation vermittelte Tonhöhenempfindung sollte sich mit dem Ort korrespondierend verändern. Folglich ist bei einer Prüfung der Elektrode in einer basalen Position, d. h. in der Nähe des Cochlea-Eingangs, eine höhere Tonhöhenempfindung zu erwarten. Unregelmäßigkeiten können auf eine von der Norm abweichende Lage des Elektrodenträgers hindeuten. In seltenen Fällen könnte ein Überschlagen der Elektrodenträgerspitze („Tip-Fold-over“) bestehen [23]. Zudem kann eine gestörte Tonotopie bei cochleären Malformationen, beispielsweise der Mondini-Dysplasie, vorliegen.

#### Untergrenze der Stimulation

Die Ermittlung der Hörschwelle bei elektrischer Stimulation stellt eine notwendige Voraussetzung für die Festlegung des Dynamikbereichs dar. Dieser Wert wird als T‑Wert oder THR-Wert bezeichnet („threshold“). Dieser Wert beschreibt eine Reizstromstärke, deren Aktivierung gerade noch keine Hörwahrnehmung oder eine gerade eben beginnende Hörempfindung erzeugt. Eine zu hohe Einstellung des Schwellenwerts kann zu Störgeräuschen führen, welche auch bei völlig ruhiger Umgebung vom Implantatträger wahrgenommen werden. Eine unzureichende Regulierung des Werts kann eine inadäquate Signalübertragung von Schallen geringer Intensität zur Folge haben, was wiederum eine Beeinträchtigung der Signalübertragung insgesamt zur Konsequenz haben kann [49].

Die zeitaufwendige Einstellung der einzelnen Elektroden kann durch den Einsatz geeigneter Methoden vereinfacht werden. In diesem Zusammenhang stehen je nach Anpassungsprogramm verschiedene Methoden der Interpolation der Werte zur Verfügung. Dazu zählen beispielsweise die simultane Stimulation von Elektrodengruppen oder die Übernahme von mittels elektrophysiologischer Verfahren ermittelten Schwellenverläufen.

#### Nutzung intraoperativer Messergebnisse als Vorlage

Einige CI-Anpassprogramme bieten die Möglichkeit, die während der Implantation bei der Durchführung von Kontrollmessungen ermittelten elektrophysiologischen Schwellen darzustellen. Bei Vorliegen dieser Daten kann eine Analyse des Schwellenverlaufs über den einzelnen Elektroden des Elektrodenträgers Aufschluss über die erforderliche Einstellung geben, was sich besonders bei der Anpassung von Kleinkindern als hilfreich erweist (s. Abschnitt „CI-Anpassung auf der Basis objektiver Messungen“). Außerdem können diese als Plausibilitätskontrolle der subjektiven Einstellung, insbesondere vor der ersten Aktivierung der Prozessormikrofone, dienen.

#### Erste Aktivierung der Prozessormikrofone

Bei der ersten Aktivierung der Prozessormikrofone (Live-Modus) werden alle Signalvorverarbeitungsoptionen zunächst deaktiviert, um den Dynamikbereich der Stimulation sicher festzulegen. Die Stimulation wird zu Beginn reduziert und schrittweise angepasst, bis eine angenehme Lautstärke für Sprache erreicht wird.

Patienten beschreiben anfangs oft einen metallischen, roboterhaften Klang, können jedoch meist bereits erste Wörter erkennen. Bei nutzbarem Resthörvermögen am Gegenohr wird empfohlen, das Hörgerät auszuschalten oder bei Patienten mit einseitiger Taubheit das normalhörende Ohr zu verschließen, um die Konzentration auf die vom CI vermittelten Höreindrücke zu fördern. Eventuell auftretendes Rauschen kann durch Verringerung der T‑Werte reduziert werden.

Patienten mit langjähriger Hochtondeprivation nehmen geräuschhafte Konsonanten zunächst oft als pfeifende Klänge wahr. Dies sollte den Betroffenen erklärt werden. Durch Akklimatisation kann in den meisten Fällen eine Normalisierung des Höreindrucks erfolgen. Selten bleiben höherfrequente Geräusche dauerhaft störend.

#### Weitere Anpassparameter

In der Mehrzahl der klinischen Fälle kann eine erfolgreiche CI-Erstanpassung anhand der herstellerspezifisch vorgegeben Stimulationsparameter durch alleinige Bestimmung der elektrischen Ober- und Untergrenzen der Stimulation erfolgen. In eher wenigen Fällen ist eine Veränderung weiterer Stimulationsparameter notwendig (s. Abschnitt „Besonderheiten und Komplikationen“).

Der am häufigsten benötigte Parameter ist die Änderung (i. d. R. die Erhöhung) der Pulsdauer zur Reduktion des Stromverbrauchs bei Erreichen der „Compliance-Grenze“ der Stromquelle oder zur Reduktion einer unerwünschten Fazialis-Kostimulation.

Weitere Parameter sind z. B. die Stimulationsrate, die Anzahl der Maxima pro Stimulationszyklus bei „n-of-m-Strategien“ (n – Anzahl gleichzeitig aktiver Elektroden pro Stimulationszyklus, m – Gesamtzahl aktivierbarer Elektroden, z. B. ACE-Strategie bei der Fa. Cochlear) und die vom akustischen Eingangssignal abhängige Funktion der elektrischen Stimulation, welche den Bereich zwischen Hörschwelle und maximaler Obergrenze abdeckt („maplaw“).

Des Weiteren bieten einige Anpassprogramme die Möglichkeit, die Trennfrequenzen der Bandpassfilterbank manuell festzulegen. Eine individuelle Berücksichtigung der patientenspezifischen Tonotopie wird dadurch ermöglicht, dass die Lage des Elektrodenträgers und der Elektrodenkontakte im Einzelnen einbezogen wird (s. Abschnitt „Anatomiebasierte Anpassung“). Es ist zu erwarten, dass dieses Vorgehen in Zukunft insbesondere bei der Anpassung von Patienten mit elektrisch-akustischer Stimulation (EAS), bei bilateralen Versorgungen und bei Patienten mit CI bei einseitiger Taubheit an Bedeutung gewinnen wird.

#### Programmierung des CI-Prozessors

Im Anschluss an die Anpassung erfolgt die Übertragung der Einstellungsparameter und die Festlegung der für den Nutzer geeigneten Signalvorverarbeitungen in den Prozessor. Hierfür stehen mehrere individuell programmierbare Programmplätze zur Verfügung, zwischen denen der Patient im Alltag bei Bedarf auswählen kann. Im Rahmen der Erstanpassung steht zunächst die Hörgewöhnung im Vordergrund, sodass auf eine umfangreiche Auswahl verschiedener Programmkonfigurationen für den Nutzer verzichtet werden sollte.

Eine möglicherweise erforderliche Synchronisation verbindet Prozessor und Fernbedienung, um nach Anlegen des Prozessors an das Implantat die Stärke der Stimulation verändern zu können. Inzwischen sind für die aktuellen Prozessormodelle Smartphone- oder teilweise sogar Smartwatch-Apps vorhanden, die eine Steuerung verschiedener Funktionen des Prozessors ermöglicht. Im Fall einer bimodalen Versorgung ist je nach Hersteller und Kompatibilität zwischen CI- und Hörgerätesystem ebenfalls eine Kopplung durchzuführen und nötigenfalls die Laufzeit anzupassen [64].

### CI-Anpassung in der Folgetherapie und Nachsorge

Grundsätzlich unterscheiden sich die Arbeitsschritte bei der Anpassung in der Folgetherapie/Nachsorge nur geringfügig von denen der Basistherapie. Auch hier muss zwingend die Magnetstärke geprüft und ggf. angepasst werden sowie eine technische Überprüfung des Implantats (Impedanzmessung) erfolgen. Außerdem ist eine regelmäßige Kontrolle der eCAP-Schwellen (s. Abschnitt „CI-Anpassung auf der Basis objektiver Messungen“) empfehlenswert.

Vor der Anpassung kommen verschiedene Verfahren der Verifikation der aktuellen Anpassung zum Einsatz (hervorzuheben ist hier die Lautheitsskalierung), um die Notwendigkeit einer Änderung der Stimulationsparameter zu prüfen und bei Bedarf weitere Maßnahmen, wie die Einleitung oder Fortführung einer therapeutischen Maßnahme, mit dem Patienten zu besprechen. Auch bei regelrechter Lautheitswachstumsfunktion sollte nicht auf eine sequenzielle Einzelkanalstimulation („Elektrodensweep“) verzichtet werden.

Darüber hinaus kann in der Folgetherapie/Nachsorge eine Anpassung der Konfiguration der Signalvorverarbeitung an die individuellen alltäglichen Anforderungen des Nutzers erfolgen. Als Ziel der Anpassung sollte ein bestmöglicher Lautheitsabgleich zwischen beiden Ohren erreicht werden (ggf. auch durch Hörgeräteanpassung des Gegenohrs bei bimodaler Versorgung) und auch das beidohrige Hören als die alltagsrelevante Situation geprüft werden (Sprachverstehen in Ruhe und im Störgeräusch, ggf. auch Richtungshören).

## CI-Anpassung auf der Basis objektiver Messungen

Während die CI-Anpassung von Erwachsenen bevorzugt basierend auf der subjektiven Rückmeldung (Lautheitsskalierung) erfolgen sollte, sind für die Anpassung bei kleinen Kindern und unkooperativen Patientinnen und Patienten objektive Verfahren, wie die Messung der elektrisch evozierten Potenziale (eCAP) des Hörnervs und der elektrisch evozierten Stapediusreflexe („electrically evoked stapedius reflex threshold“, eSRT), besonders hilfreich. Auch die Ableitung elektrisch evozierter Hirnstammpotenziale (eBERA) und kortikaler Potenziale (eCERA) kann zur Unterstützung des Anpassprozesses nützlich sein.

### Elektrisch evozierte Summenaktionspotenziale des Hörnervs

Das über die Elektroden des Implantats registrierte elektrisch evozierte Summenaktionspotenzial des Hörnervs (eCAP) ist die Spannungsänderung als Funktion der Zeit, die durch die Depolarisation der Hörnervenfasern entsteht. Das eCAP ist eine synchrone physiologische Antwort einer Gesamtheit von Hörnervenfasern auf eine elektrische Stimulation und durch ein negatives Potenzial N1, gefolgt von einem positiven Potenzial P2, gekennzeichnet. Die eCAP-Amplitude steigt mit zunehmender Reizstärke und kann Spitze-zu-Spitze-Amplituden von bis zu 1 mV betragen, liegt aber i. d. R. eher zwischen 10 und 100 µV. Das eCAP entspricht der Welle I der elektrisch evozierten auditorischen Hirnstammpotenziale.

eCAP bieten gegenüber der Ableitung von zentraleren Antworten den Vorteil, dass sie robust gegenüber Einflüssen der Narkose sind und somit auch für intraoperative Messungen eingesetzt werden können. Darüber hinaus ist die Messung aufgrund der Nutzung intracochleärer Elektroden robust gegen die myogene Aktivität des Patienten (d. h. potenzielle Artefakte durch Muskelaktivität), sodass Patienten während der eCAP-Messungen nicht still liegen, schlafen oder sediert werden müssen. Durch die Messung im Nahfeld des Hörnervs sind für eCAP-Messungen nur wenige Mittelungen erforderlich, sodass die Messdauer wesentlich kürzer ist als für Hirnstamm- oder kortikale Messungen. Außerdem sind eCAP bereits im ersten Lebensjahr robust messbar und viel weniger von Reifungseffekten beeinflusst als kortikale Antworten.

**Abb. 3:**
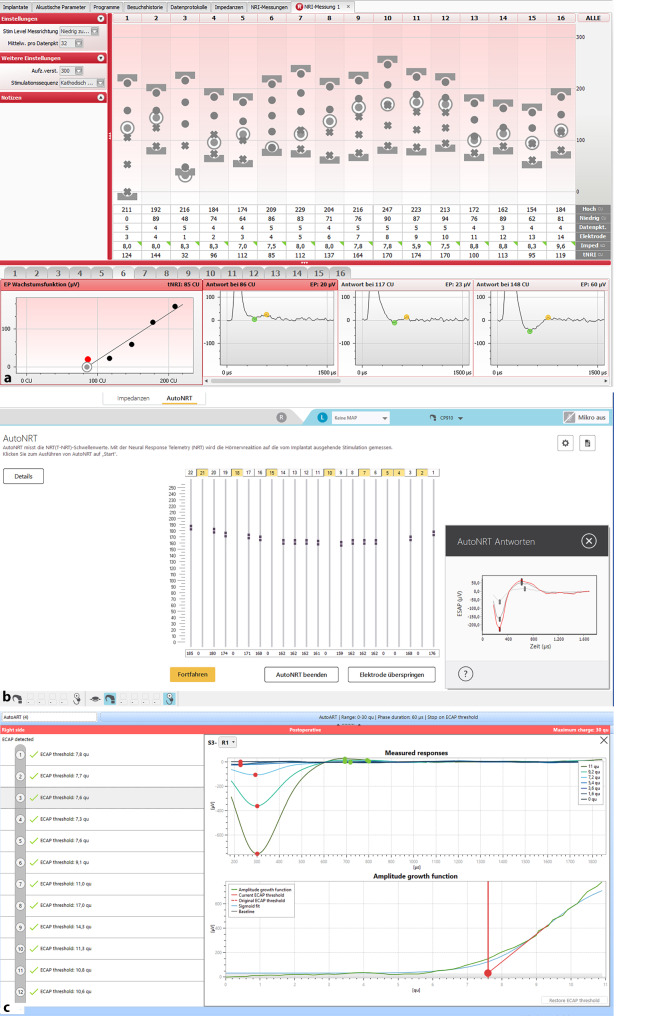
Herstellerspezifische Messroutinen zur automatischen Bestimmung von eCAP-Schwellen. **a** Advanced Bionics NRI; **b** Cochlear AutoNRT, **c** MED-EL AutoART. *eCAP* elektrisch evozierte Summenaktionspotenziale des Hörnervs

Der elektrodenspezifische Schwellenwert des eCAP kann mit den durch die Implantathersteller bereitgestellten Anpassprogrammen (Abb. 3) automatisiert gemessen werden. Die Hersteller Advanced Bionics (Bezeichnung der ermittelten Schwelle: t-NRI [Threshold-Neural Response Imaging], Abb. 3a) und Cochlear (Bezeichnung der ermittelten Schwelle: T-NRT [Threshold-Neural Response Telemetry], Abb. 3b) nutzen hierfür das Verfahren der linearen Interpolation/Regression diskreter Messpunkte mit mehreren Messwiederholungen. Der Hersteller MED-EL hat vor einigen Jahren das Verfahren „AutoART“ vorgestellt (Abb. 3c), wo die Anzahl der Messwiederholungen je Amplitude zugunsten einer feineren Amplitudenauflösung reduziert wurde. Für spezielle Fragestellungen (z. B. zur Bestimmung der Amplitudenwachstumsfunktion, „spread of excitation“ usw.) oder bei der Abwesenheit von eCAP in der automatisierten Messung bieten die Hersteller Expertenmodi an, die eine individualisierte Parametrisierung der Messung ermöglichen (z. B. Variation der Stimulationsrate, Pulsdauer usw.).

eCAP-Schwellenwerte können zur Unterstützung bei der Einstellung von elektrischen Stimulationspegeln verwendet werden, wenn keine behaviorale Anpassung möglich ist. Hierfür sollten vorab Messungen der eCAP-Schwelle an möglichst allen Elektroden vorliegen. Bei dem Einsatz von eCAP-Schwellen für die CI-Anpassung ist zu bedenken, dass die ermittelte Schwelle u. a. abhängig von der Stimulationsrate und der Pulsdauer ist. In der Regel entsprechen die Stimulationsparameter bei der Messung nicht den klinischen Stimulationsparametern (weder unterer noch oberer Stimulationspegel) der CI-Programmierung. So lässt sich beispielsweise eine tendenzielle Verschlechterung der Korrelation zwischen eCAP-Schwellenwerten und einer klinischen CI-Programmierung mit sehr hohen Stimulationsraten beobachten [35]. Die eCAP-Schwellenwerte liegen üblicherweise deutlich über den subjektiv ermittelten elektrischen Hörschwellen und häufig innerhalb des oberen Drittels des elektrischen Dynamikbereichs. Das eCAP-Schwellenprofil ähnelt häufig dem Profil von T(HR)- oder C/M(CL)-Werten.

Eine optimale Bestimmung des elektrischen Dynamikbereichs ist mit dieser Methode nicht möglich, sodass die behaviorale Anpassung wenn möglich immer zu bevorzugen ist. Eine Kombination mit einzelnen subjektiv bestimmten Schwellenwerten und/oder mit anderen objektiven Verfahren kann die Genauigkeit der Anpassung weiter verbessern.

### Elektrisch evozierter Stapediusreflex

Eine weitere objektive Methode für die CI-Anpassung ist die Messung elektrisch ausgelöster Stapediusreflexe (eSRT). Diese können zum einen verwendet werden, um eine grundsätzliche Funktion des Implantatsystems und der Hörnervfunktion bis auf Hirnstammebene zu prüfen. Außerdem sind eSRT insbesondere zur Bestimmung des elektrodenspezifischen maximalen Stimulationspegels und zur Vermeidung/Kontrolle einer möglichen Überstimulation äußerst hilfreich und damit zur Anpassung bei Kindern gut geeignet. Das Verfahren der eSRT-basierten CI-Anpassung ist in der klinischen Routine jedoch deutlich aufwendiger und daher weniger verbreitet, da für die Aufzeichnung der eSRT im Anpassraum ein (im Idealfall mit Triggersignal synchronisiertes) Tympanometer benötigt wird. Alternativ kann die in klinischen Tympanometern häufig verfügbare Funktion der Langzeitimpedanzmessung („reflex decay“) verwendet werden.

Vor Durchführung der eSRT-basierten Anpassung ist zwingend zu prüfen, ob bei dem für die Messung verwendete Ohr eine normale Mittelohrfunktion (Typ-A-Tympanogramm) vorliegt. Da der Stapediusreflex unabhängig von der Stimulationsseite ipsilateral und kontralateral ausgelöst wird, kann das zu messende Ohr prinzipiell frei gewählt werden. In der Literatur wurde häufig das kontralaterale Ohr empfohlen [32, 53], aber auch Messungen auf dem ipsilateralen mit CI versorgten Ohr sind möglich [21].

Ein Vorteil gegenüber der eCAP-basierten Anpassung ist, dass der verwendete elektrische Stimulus üblicherweise dem der klinischen Anpassung entspricht. Daher gibt es eine starke Korrelation zwischen eSRT-Schwellen und den MCL-/C-Werten der klinischen Anpassung [53]. Alternativ ist auch eine Stimulation im Freifeld im „Live-Modus“ des Anpassprogramms möglich [21]. Zur Vermeidung einer Überstimulation erfolgt die Schwellenbestimmung zunächst mit nur geringem Stimulationspegel und wird sukzessive bis zur Detektion eines deutlichen Stapediusreflexes erhöht. Anschließend erfolgt die Pegelreduktion mit einer geringeren Schrittweite, bis keine Reaktion mehr vorhanden ist.

Bei dem Einsatz der eSRT für die CI-Anpassung ist zu berücksichtigen, dass unabhängig von der intakten Funktion des CI-Systems und des Hörnervs nicht in allen Fällen (nur etwa 60–80 %) eine sichere Bestimmung einer eSRT möglich ist. Darüber hinaus muss der Patient sich während der gesamten Messung möglichst ruhig verhalten, um keine Bewegungsartefakte und akustischen Störsignale zu erzeugen.

Da eine zusätzliche eCAP-Messung i. d. R. schnell und mit hoher Akzeptanz möglich ist, kann die Kombination von eSRT- und eCAP-Messungen zu einer Verbesserung der Genauigkeit der klinischen Anpassung führen [62].

### Elektrisch evozierte Hirnstammpotenziale

Die Ableitung elektrisch evozierter Hirnstammpotenziale (eBERA) ist ein häufig eingesetztes Verfahren bei unklarer Integrität des Hörnervs wie z. B. bei Patienten mit Vestibularisschwannom oder bei postoperativ unerwartet schlechtem Ergebnis mit unauffälligen eCAP-Schwellen. Hierfür ist, wie auch bei der eCERA, ein mittels Triggersignal mit dem CI-System synchronisiertes Messsystem für EEG-Signale notwendig. Empfehlungen zur Parametrisierung der elektrischen Stimuli sind in der Arbeit von Baljic et al. [6] und Dziemba et al. [16] dargestellt.

Da die Stimulationsparameter der CI-Systeme (z. B. Empfehlung bipolare Stimulation bei Cochlear oder simultane Stimulation benachbarter Kanäle bei MED-EL [6]) erheblich von der klinischen Stimulation abweichen und bisher nur wenige Referenzdaten vorliegen, ist die eBERA als Ersatz oder Ergänzung einer subjektiven/behavioralen Anpassung derzeit noch ungeeignet.

### Elektrisch evozierte kortikale Potenziale

Die Messung elektrisch evozierter kortikaler Potenziale (eCERA) kann sich in der Phase nach bereits erfolgter Erstanpassung insbesondere bei Kindern für die Optimierung der CI-Anpassung eignen. Kortikale Potenziale sind (späte) ereigniskorrelierte Potenziale und repräsentieren daher die eher bewusste Schallwahrnehmung bzw. -verarbeitung. Die Reizantwort beinhaltet mehrere Potenzialkomplexe, wobei sich die Auswertung des P1-N1-P2-Komplexes für die CI-Anpassung als am nützlichsten erwiesen hat.

Der Einsatz von Freifelddarbietung für die CERA-Messung wurde erfolgreich für die Anpassung von Hörgeräten bei Kleinkindern evaluiert [55]. Es können beispielsweise synthetische oder natürliche Sprachstimuli unterschiedlicher Frequenzbereiche eingesetzt werden. Bei stark asymmetrischem Hörverlust, also im Fall einer CI-Versorgung mit nur gering ausgeprägter Schwerhörigkeit oder gar Normalhörigkeit, kann das kontralaterale Ohr jedoch nicht ausreichend maskiert werden. Hier ist alternativ eine direkte elektrische Stimulation über das CI möglich [54].

Deniz et al. [13] konnten keine signifikanten Unterschiede zwischen mittels des Verfahrens der eSRT und mittels des Verfahrens der eCERA ermittelten MCL-Werte feststellen. Zur Schätzung der MCL-Werte mittels eCERA ist jedoch eine Rückmeldung des Patienten über die subjektive Lautheit und/oder eine aufmerksame Beobachtung des Patienten vonnöten.

## Signalvorverarbeitung

Unabhängig von den durch Einzelkanalstimulation bestimmten Ober- und Untergrenzen der elektrischen Stimulation kommen im „front-end“ des CI-Systems (d. h. in dem CI-Prozessor) verschiedene Verfahren zum Einsatz, um der fehlenden Funktion des Innenohrs sowie den prinzipbedingten Einschränkungen der elektrischen Stimulation des Hörnervs Rechnung zu tragen. Dies betrifft insbesondere den eingeschränkten Dynamikbereich und die mit der geringen Frequenzauflösung und fehlenden zeitlichen Feinstruktur einhergehende schlechtere Trennbarkeit auditorischer Objekte. Letzteres macht sich insbesondere durch ein deutlich schlechteres Sprachverstehen im Störgeräusch im Vergleich zu Normalhörenden bemerkbar.

### Automatische Pegelanpassung und Dynamikkompression

Zur Optimierung des Dynamikbereichs werden automatische (i. d. R. mehrstufige) Verstärkungssteuerungen („automatic gain control“, AGC) bzw. Dynamikkompression und ggf. -expansion mit unterschiedlichen Zeitkonstanten eingesetzt, um kurzzeitige Transienten (Plosive in Sprachsignalen, „Türzuschlagen“ usw.) abzufangen, abrupte Pegelsprünge zu reduzieren und im Langzeitsignalverlauf den akustischen Dynamikbereich optimal auf die elektrische Stimulation anzupassen. Während das Verhalten der AGC bei den meisten Herstellern nicht regelbar ist, kann das Kompressionsverhalten häufig individuell angepasst werden. Hier ist zu beachten, dass die Kompressionsrate aufgrund des geringen elektrischen Dynamikbereichs des Hörnervs üblicherweise höher ist als bei Hörgerätesystemen. Im Rahmen der CI-Anpassung ist in der Mehrzahl der Fälle keine Änderung an den vom Hersteller vorgegeben Parametern notwendig. Bei einer Langzeitertaubung kann bei einer hohen Empfindlichkeit des Patienten und einer flachen Lautheitswachstumsfunktion im Einzelfall eine Anpassung der Kompression oder anderer Parameter des Dynamikbereichs (z. B. T‑SPL-Werte, C‑SPL-Werte, und „maplaw“) nützlich sein.

Bei der Versorgung von Kleinkindern sollte nach Auffassung der Autoren auf die Aktivierung zusätzlicher „Komfortfunktionen“ wie z. B. der automatischen Transientenreduktion verzichtet werden, um in der kindlichen Hörentwicklung eine möglichst realistische Wahrnehmung alltäglicher Geräusche zu ermöglichen.

### Störgeräuschunterdrückung und Richtmikrofone

Während bei heutiger CI-Versorgung meist ein gutes bis sehr gutes Sprachverstehen in Ruhe erreicht wird, bleibt das Sprachverstehen im Störgeräusch im Vergleich zu Normalhörenden weiterhin sehr stark beeinträchtigt. Daher besteht eine hohe Notwendigkeit, in alltäglichen Störgeräuschsituationen durch geeignete Signalvorverarbeitung eine bestmögliche Übertragung von Sprachsignalen zu ermöglichen. Hierfür gibt es 2 Ansätze:

- die einkanalige Störgeräuschunterdrückung und
- der Einsatz von (adaptiven) Richtmikrofonen („beamformer“).

Beide Ansätze wurden aus der Hörgerätetechnik übernommen und bei Bedarf für die Anforderungen an das CI-System neu parametrisiert.

Bei der einkanaligen Störgeräuschunterdrückung (firmenspezifische Bezeichnungen: Advanced Bionics [AB] „ClearVoice“, Cochlear „SNR-NR“, MED-EL „ANR“) wird das Eingangssignal in mehrere Frequenzbänder unterteilt und der Signal-Rausch-Abstand („signal-to-noise ratio“, SNR) geschätzt. Bänder mit geringerem SNR werden in der Verstärkung reduziert. Diese Algorithmen funktionieren i. d. R. recht stabil für eher stationäre Störgeräusche, während bei modulierten und insbesondere sprachartigen Störgeräuschen keine Verbesserung zu erwarten ist.

Zur räumlichen Störschallunterdrückung durch Richtmikrofone kann die Richtcharakteristik und damit der räumliche Fokus durch digitale Signalverarbeitung (Laufzeitvariation und Pegelgewichtung von mindestens 2 Mikrofoneingangssignalen) variiert werden. Als Standardrichtcharakteristik ist für die CI-Anpassung im Rahmen der Basistherapie eine Richtcharakteristik ähnlich der Ohrmuscheleigenschaften empfehlenswert (firmenspezifische Bezeichnungen: AB „Real Ear Sound“ oder T‑Mic, Cochlear „Standard“ entsprechend einer Subniere, MED-EL Mikrofonmodus „Natürlich“). Zusätzlich werden je nach Hersteller z. B. Richtcharakteristiken mit omnidirektionaler Richtcharakteristik oder maximaler Reduktion verschiedener Winkel im hinteren Halbraum (Hyperniere oder Superniere) angeboten. Bei den adaptiven Algorithmen wird die Position von Sprache und Störgeräusch geschätzt und die maximale räumliche Unterdrückung (Nullstelle des Polardiagramms) entsprechend der Störgeräuschposition optimiert (firmenspezifische Bezeichnungen: AB „UltraZoom“, Cochlear und MED-EL „Adaptiv“) [58, 60, 61]. Teilweise ist auch die bilaterale Kombination der Sprachprozessoren zu einem binauralen Beamformer (AB „StereoZoom“) oder die Kombination aus räumlicher Signalverarbeitung und nachgeschalteten digitalen Filtern (Cochlear „ForwardFocus“) verfügbar. In realen Hörsituationen mit wechselnden oder sich bewegenden Nutz- und Störschallquellen sowie im Nachhall ist die SNR-Verbesserung durch Richtmikrofone deutlich geringer als in Laborstudien mit einzelnen Störgeräuschquellen unter Freifeldbedingungen [18].

Insbesondere bei der Verwendung besonders starker Störgeräuschreduktionstufen oder besonders stark bündelnder Richtmikrofone ist je nach Hörsituation eine Verschlechterung der Klangqualität, Beeinträchtigung der Lokalisationsfähigkeit und ein Überhören von potenziellen Warnsignalen (z. B. Kfz-Geräusche im Straßenverkehr) nicht auszuschließen. Bei der Anpassung der CI-Systeme hinsichtlich der Signalvorverarbeitungen sollten daher im Rahmen von Basistherapie, Folgetherapie und Nachsorge die individuellen Bedürfnisse des Patienten regelmäßig erfragt und die Bereitstellung der entsprechenden Funktionen erläutert werden. Alternativ oder zusätzlich kommt die Aktivierung eines automatischen Programms in Frage (s. folgender Abschnitt), wo der Eingriff in die Signalvorverarbeitung häufig nur mit moderater Parametrisierung erfolgt.

Im Rahmen der Anpassung bei Kindern sollte auf eine zu stark störgeräuschreduzierende Signalvorverarbeitung oder adaptive Beamformer möglichst verzichtet werden, um in der kindlichen Hörentwicklung eine realistische Wahrnehmung alltäglicher Geräusche und eine bestmögliche Entwicklung des Richtungshörens zu ermöglichen.

### Automatikprogramme

Ähnlich wie bei der Nutzung einer Sehhilfe besteht auch bei Nutzern von Hörhilfen häufig der Wunsch, das Hörsystem zu Beginn des Tages aufzusetzen und am Ende des Tages abzunehmen, ohne sich in Abhängigkeit von der gerade vorherrschenden Hörsituation bewusst damit befassen zu müssen. Da für den bestmöglichen Hörerfolg je nach Hörsituation eine unterschiedliche Konfiguration der Signalvorverarbeitung notwendig sein kann, muss in diesem Fall eine automatisierte Szenenerkennung und Umstellung der entsprechenden Parameter erfolgen. Die Szenenerkennung basiert auf Klassifikatoren/Mustern für die häufigsten alltäglichen Hörsituationen (z. B. Ruhe, Störgeräusch, Sprache in Ruhe, Sprache im Störgeräusch, Musik, Wind usw.), die anhand entsprechender akustischer Merkmale aus Trainingsmaterial detektiert werden (firmenspezifische Bezeichnungen: AB „Auto Sense“, Cochlear „SCAN“, MED-EL „Adaptive Intelligence“). Damit der Nutzer die Umschaltprozesse nicht als störend empfindet, muss die Klassifikation und die Umschaltung der Signalvorverarbeitung eine ausreichende Trägheit besitzen (üblicherweise im unteren bis mittleren zweistelligen Sekundenbereich).

Je nach Alter, Technikaffinität und Komplexität der alltäglichen Hörumgebungen ist die Verwendung der automatischen Algorithmen für verschiedene Personengruppen unterschiedlich empfehlenswert und sollte individuell im Rahmen der CI-Anpassung mit den Nutzern besprochen werden. Bei Kleinkindern sollte zunächst auf automatische Szenenerkennung verzichtet werden. Ebenso sollten automatische Szenenerkennungen während der Durchführung der kategorialen Lautheitsskalierung und der Anpassung der unteren und oberen Schwellenwerte im Live-Modus deaktiviert sein.

## Anatomiebasierte Anpassung

Obwohl die Anwendung des CI große Vorteile bieten kann, schildern einige Nutzer das Hören mit einem CI als unangenehm und manchmal störend, insbesondere in der Anfangsphase. Die mit dem CI erzielten Hörleistungen zeigen große interindividuelle Unterschiede, die selbst nach einer längeren Eingewöhnungszeit verbleiben. In realen Hörsituationen mit Hintergrundgeräuschen stellt sich die CI-vermittelte Sprachverständlichkeit oft unzureichend dar [50]. Ein möglicher Grund für die eingeschränkte Hörqualität könnte die vom Hersteller vorgegebene Zuordnung der Mittenfrequenzen der Filterbank des Audioprozessors sein, die für alle Patienten gleich ist [42]. Bei der Erstaktivierung des Audioprozessors wird i. d. R. eine Standardfrequenzzuweisung eingestellt, ohne die individuelle Anatomie der Cochlea und die genaue Position der Elektroden zu berücksichtigen. Die Länge des cochleären Gangs („cochlear duct length“, CDL) und die Einführtiefe der Elektroden sind jedoch individuell verschieden, sodass die elektrischen Frequenzbänder an einer bestimmten intracochleären Position um bis zu 2 Oktaven von der physiologischen Frequenzzuweisung abweichen können. Verschiedene, z. T. noch laufende Studien, untersuchen, ob eine an die individuelle Position der Elektroden angepasste anatomiebasierte Anpassung („anatomy-based fitting“, ABF) die Klangqualität und die Sprachwahrnehmung im Vergleich zur konventionellen Frequenzverteilung verbessern kann.

### Erstellung der ABF

**Abb. 4:**
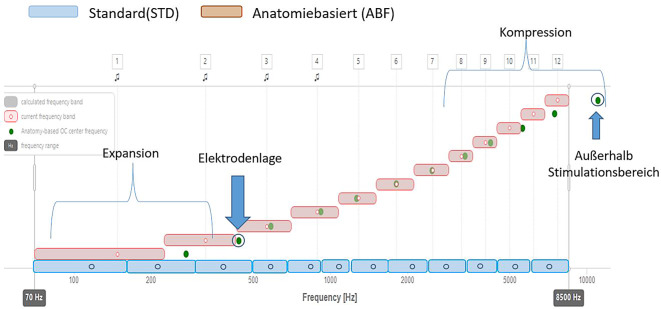
Schematische Darstellung der Verteilung der Filterfrequenzbereiche (nach [25]).Anatomiebasierte Anpassung der Filterfrequenzen (FF) nach den Vorgaben des Herstellers MED-EL. *Grüne Punkte *anhand der Bildgebung ermittelte, zur Elektrodenlage korrespondierende Frequenz. *Blau *Standard-FF-Verteilung. *Rot umrandet *anatomiebasierte FF-Verteilung. Im apikalen Bereich Expansion, im basalen Bereich Kompression der FF-Verteilung. Elektrode 12 außerhalb des vom Sprachprozessor abbildbaren Frequenzbereichs

Zur Erstellung einer anatomiebezogenen Einstellung (ABF) sind i. d. R. sowohl prä- als auch postoperative Bildgebungsuntersuchungen erforderlich. Die präoperativ mittels Computertomographie (CT) oder digitaler Volumentomographie (DVT) erstellten Schichtaufnahmen werden mit einem geeigneten Rekonstruktionsverfahren, bspw. OTOPLAN® (Fa. CASCINATION AG, Bern, Schweiz) zur Berechnung der CDL und zur Modellierung der erwarteten Einführtiefen der Elektrode verwendet, um das Design (Länge) der zu verwendenden Elektrode zu bestimmen. Anhand von postoperativ erstellten Schichtbildern wird die Position der einzelnen Elektrodenkontakte innerhalb der Cochlea durch das entsprechend ausgelegte Rekonstruktionsprogramm bestimmt. Beispielsweise berechnet OTOPLAN die Mittenfrequenzen der Bandpassfilter anhand der Position der Elektroden nach den Formeln von Alexiades et al. [1], Escudé et al. [19] und Greenwood [27] (Details in [26]). Da die tonotope Frequenzverteilung in der Cochlea nicht linear, sondern eher logarithmisch zu höheren Frequenzen hin verläuft, wie dies in der Greenwood-Funktion zum Ausdruck kommt, basiert die vom Hersteller vorgegebene Zuordnung der Filtermittenfrequenzen hauptsächlich auf einer logarithmischen Verteilung der Bandfilter-Mittenfrequenzen. Im basalen Bereich der Cochlea liegen Hersteller- und ABF-Zuordnungen daher sehr nahe beieinander. Die entsprechende Mittenfrequenz kann in die klinische Anpasssoftware (bspw. MAESTRO, ab Version 9.0.5, MED-EL) importiert werden. Die von OTOPLAN vorgegebene tonotope Zuordnung des Corti-Organs („organ of Corti“, OC) wird dann als Mittenfrequenz verwendet. Bei weniger tiefen Insertionen werden im tieferen Frequenzbereich Mittenfrequenzen expandiert. Bedingt durch die Begrenzung des oberen Frequenzbereichs des CI-Prozessors (gegenwärtig etwa 8500 Hz) werden die Bandbreiten der Filterbänke in der basalen Region komprimiert, wenn Elektroden im basalen Bereich oberhalb dieser Frequenz liegen. Diese Zuordnung stellt sicher, dass die Filterbänke im medialen Bereich der Cochlea den tonotopen Mittenfrequenzen innerhalb der Cochlea entsprechen (Abb. 4). Alternative Vorschläge für eine individuelle Frequenzzuordnung versuchen, im apikalen und basalen Bereich die Zuordnung der Frequenzbänder näher an die berechneten OC-Mittenfrequenzen zu bringen. Hierbei wird die untere Grenzfrequenz des apikalsten Elektrodenkontakts (E1) erhöht, um die niedrigste Mittenfrequenz in Richtung der berechneten OC-Frequenz zu verschieben. Liegt die errechnete OC-Frequenz oberhalb des Übertragungsbereichs des CI-Prozessors, wird die basalste Elektrode abgeschaltet, um eine bessere Übereinstimmung der Frequenzzuordnung zu erreichen [25].

### Ergebnisse der ABF

Zwischenergebnisse einer laufenden Studie deuten darauf hin, dass ABF die Hörqualität von CI-Trägern verbessern kann, ohne das Sprachverstehen in Ruhe zu beeinträchtigen [25]. Es zeigte sich ein Trend zur Verbesserung der Hörqualität mit ABF. Allerdings wurde bei einigen Probanden ein Akzeptanzproblem bei der Umstellung der Frequenzzuordnung beobachtet. Die Änderung der Frequenzzuordnung kann bei CI-Trägern eine längere Gewöhnungszeit erfordern.

## Besonderheiten und Komplikationen

Die CI-Anpassung kann mit verschiedenen Besonderheiten, Komplikationen und Herausforderungen verbunden sein. Diese können medizinische, technische, rehabilitative und psychosoziale Aspekte umfassen, die Einflüsse auf die Einstellung des CI-Systems haben können.

### Infobox 1 Besonderheiten und Komplikationen mit möglichen Auswirkungen auf die CI-Anpassung

Medizinisch

- Restgehörerhalt (elektrisch-akustische Stimulation, EAS; hybride Stimulation)
- Entzündungen oder Infektionen
- Wundheilungsstörungen
- Abstoßung des Implantats
- Schwindel oder Gleichgewichtsprobleme
- Verstärkung oder Neuauftreten von Tinnitus
- Fazialisparese (in Begleitung mit Hörnervdefekt)

Technisch

- Einstellung des Prozessors: Besonderheiten bei der optimalen Anpassung der Stimulationsparameter
- Gerätestörungen: Probleme mit der internen oder externen Hardware.
- Interferenz: mögliche Beeinflussung der Funktion durch elektronische oder magnetische Felder
- Elektrodenplatzierung: partielle Insertion, Tip-Fold-over, Wechsel intracochleärer Skalen

Rehabilitativ

- Gewöhnung an das Implantat: Klang anfangs oft als ungewohnt oder künstlich empfunden
- Hörtraining: unzureichende oder fehlende Rehabilitation/Hörtherapie
- Individuelle Variabilität: nicht von jedem Patienten die gleichen Hörfähigkeiten erreichbar
- Motivationsprobleme: Entmutigung der Patienten durch einen langsamen Fortschritt möglich

Psychosozial

- Erwartungsmanagement: durch unrealistische Erwartungen an das Implantat möglicherweise Frustration
- Isolation oder Stress: durch Anpassung an die CI-Stimulation psychische Belastung möglich
- Soziale Akzeptanz: Furcht von Patienten vor Mobbing durch das Tragen des externen Prozessors

Langzeitprobleme

- Migration: Verschiebung des Stimulators im Gewebe möglich, ggf. Dislozierung des Elektrodenarrays aus der Cochlea
- Progression des Hörverlusts: Fortschreiten des Hörverlusts auf der Gegenseite oder bei Erhalt des Restgehörs im implantierten Ohr möglich
- Notwendigkeit von Revisionseingriffen: Austausch oder des Implantats bei technischen Problemen, Reinsertion des Elektrodenarrays bei Dislokation

Innerhalb eines Zeitraums von 10 Jahren war nach den Ergebnissen einer retrospektiven Studie jeder zweite Patient von einem unerwünschten Ereignis in Zusammenhang mit seiner CI-Versorgung betroffen [37, 39], wobei Schmerzen im Implantatbereich, Störungen der Gleichgewichtsfunktion, Infektionen und Beeinträchtigungen der Hörqualität den größten Anteil aufwiesen.

Die erfolgreiche Anpassung eines CI erfordert daher ein multidisziplinäres Team aus HNO-Ärzten, Audiologen, Logopäden und Psychologen, um diese Herausforderungen zu bewältigen und den Patienten optimal zu unterstützen.

### Unerwünschte Kostimulationen

Bei der Festlegung der zulässigen Stimulationsstärke obliegt es dem CI-spezialisierten Audiologen sicherzustellen, dass keine unbeabsichtigte Mitstimulation des Gesichtsnervs erfolgt. Eine derartige Stimulation kann eine dezent ausgeprägte Kontraktion der Gesichtsmuskulatur im Bereich des Augenlids, des äußeren Augenwinkels (Angulus oculi lateralis), des Mundwinkels (Angulus oris) oder der Wange zur Folge haben. In einigen Fällen bleiben diese Kontraktionen für den Patienten jedoch subjektiv unbemerkt. Bei Patienten, bei denen cochleäre Malformationen, Meningitis, fortgeschrittene Otosklerose oder Sensibilitätsstörungen des Hörnervs diagnostiziert wurden, manifestieren sich derartige Nebenwirkungen vergleichsweise häufiger als bei Patienten mit anderen Ursachen einer Hörminderung.

Bei einer starken Kostimulation des Gesichtsnervs, die nicht durch eine Umprogrammierung (Deaktivierung betreffender Elektroden, Erhöhung der Pulsweite, falls möglich: Veränderung der Referenzelektrode) beherrscht werden kann, ist wenn möglich ein Wechsel der Pulsform (triphasische Pulse, [5]) oder des Implantats zu empfehlen, wenn durch das neue Modell eine nebenwirkungsfreie Stimulation des Hörnervs erwartet werden kann [24]. Patienten mit einer fortschreitenden Otosklerose als Ursache für die Hörminderung sind häufiger als andere Ätiologiegruppen von Kostimulationen des Gesichtsnervs betroffen.

### Tip-Fold-over

In seltenen Fällen kann sich ein Umklappen der Elektrodenspitze bei der Einführung des Elektrodenarrays in die Cochlea ereignen [23]. Falls dies intraoperativ nicht bemerkt wird, sollte anhand einer postoperativ erstellten CT- oder DVT-Untersuchung in Verbindung mit einer Feldimpedanzmessung durch Analyse der Matrix des elektrischen Spannungsfelds diejenige Elektrode identifiziert werden, bei welcher der Umschlagpunkt auftritt. Elektroden, die sich apikal der Umschlagstelle befinden, sollten deaktiviert werden, um eine Überlagerung der elektrischen Felder von unterschiedlichen Kanälen zu vermeiden und somit eine Verbesserung der Hörqualität zu erreichen.

### Elektrodenfehllage und Elektrodenmigration

Patienten, mit Elektrodenmigration (EM) oder unvollständig eingeführtem Elektrodenarray können durch audiologische und elektrophysiologische Tests identifiziert werden. Im Allgemeinen stellt sich bei einer deutlichen Verschlechterung der Sprachwahrnehmung oder Sprachqualität der Verdacht auf EM, und weitere diagnostische Verfahren zur Überprüfung der Elektrodenmigration sollten dann eingeleitet werden.

#### Infobox 2 Testverfahren bei Verdacht auf Elektrodenmigration. (Nach [48])

- Sprachwahrnehmung in Ruhe und bei Störgeräuschen: verminderte Hörleistung
- Aufblähkurve: deutlich erhöhte Schwellen bei 6–8 kHz
- Elektrodenimpedanzen: deutliche Erhöhung bei basalen Elektroden
- Elektrisch evozierte Summenaktionspotenziale (eCAP): bei basalen Elektroden nicht messbar
- eCAP-Profil: deutliche Verschiebung in Richtung apikaler Elektroden im Vergleich zu den initialen Messungen
- Einzelelektrodenstimulation: durch basale Elektroden keine Erzeugung einer Hörempfindung oder nichtauditive Effekte (z. B. Stimulation des Gesichtsnervs)
- bilaterale Tonhöhenbewertung (bei bilateralen CI-Nutzern): Tonhöhenverschiebung in Richtung basaler Elektroden
- T- (Schwellenwert) und C‑ (angenehme Lautstärke) Profile: deutliche Verschiebung der „fitting map“ in Richtung apikaler Elektroden

Elektroden, die keine Hörwahrnehmung oder einen inadäquaten Lautheitsaufbau zeigen, sollten deaktiviert werden. Bei unzureichendem Hörerfolg sollte eine Revision mit Reinsertion des Elektrodenarrays verfolgt werden. Hierbei sollte eine ausreichende Fixierung der Elektrodenzuleitung vorgenommen werden [43].

### Elektrodenfehler

In seltenen Fällen können sich Fehler der Kontaktierung der Platinelektroden im Fertigungsprozess des Implantats oder Kabelbrüche bei der mechanischen Manipulation des Elektrodenarrays während der Operation ergeben. Zur Erkennung derartiger Fehler ist die telemetrische Prüfung der Elektrodenimpedanzen hilfreich, die auch bereits vor der Verwendung des Implantats durchgeführt werden kann [40]. Ergeben sich Hinweise für einen Kurzschluss zwischen 2 Elektrodenkontakten, muss abgewogen werden, ob eine Deaktivierung beider Elektroden vorgenommen werden muss. Dies ist bei nicht direkt benachbarten Elektroden empfehlenswert, da sich ansonsten ungünstige, nicht tonotope Überlagerungen der Stimulationsfelder ergeben. Bei einer Unterbrechung der Zuleitung zeigen die Ergebnisse der Telemetrie eine sehr hohe Elektrodenimpedanz mit ausbleibender Hörempfindung oder fehlendem Lautheitsaufbau in der Einzelkanal-Stimulation. Eine Deaktivierung der betroffenen Elektroden ist erforderlich, um den spektralen Anteil des Eingangssignals im vom Kurzschluss betroffenen Kanal auf die Nachbarelektroden zu verteilen.

### Schwankungen und Anwachsen der Elektrodenimpedanz

Veränderungen des elektrischen Widerstands der Elektrodenkontakte können durch folgende Prozesse bewirkt werden:

- Veränderungen der Leitfähigkeit der Lymphflüssigkeit in den cochleären Skalen
- Fibrosierung der Elektrodenkontaktoberfläche (z. B. nach längerer Nichtnutzung des CI)
- Bei monopolarer Stimulation: Veränderungen im Umfeld der Referenzelektrode (z. B. Entstehung einer Luftblase durch Schnäuzen oder ossäre Neubildungen)
- Intracochleärer Skleroseherd
- Veränderungen der Leitfähigkeit des Felsenbeins (Os petrosum) durch Otospongiose
- Instabile Verbindung/Kontaktierung der Elektroden

Da der für einen bestimmten Lautheitseindruck erforderliche Stimulationsstrom vom Widerstand der jeweiligen Elektrode abhängt, führen Schwankungen der Elektrodenimpedanz zu veränderten Höreindrücken, da der Stimulationsstrom i. d. R. nicht angepasst wird. Ein Nachführen der Stimulationsgrenzen oder die Erhöhung der Pulsweite sorgt in vielen Fällen für eine Behebung des Problems.

Alle Patienten werden nach einer CI-Operation darauf hingewiesen, dass Schnäuzen der Nase für einen längeren Zeitraum zu unterlassen. Ebenso ist das Heben von schweren Lasten nicht gestattet. Hintergrund ist, dass sich durch den Aufbau eines Überdrucks im Mittelohrbereich durch die möglicherweise noch nicht komplett mit Bindegewebe eingeheilte Elektrodenzuleitung eine mit Luft gefüllte Tasche im Bereich des Implantatlagers bilden kann. Da sich bei vielen Implantaten im Bereich des Stimulatorgehäuses eine Referenzelektrode befindet, kann dieses Luftpolster eine massive Erhöhung bis hin zur Unterbrechung der Leitfähigkeit dieses Massekontakts bewirken. Wenn sich bei Druck auf das Implantat die Hörempfindung deutlich ändert und/oder Veränderungen der Elektrodenimpedanz nachgewiesen werden können, ist als Ursache das Luftpolster über der Referenzelektrode anzunehmen. Abhilfe in solchen Fällen kann durch einen Druckverband über dem Implantat und konsequente Umsetzung des Schnäuzverbots geschaffen werden [38].

Kommt es in seltenen Fällen im Rahmen einer intracochleären Sklerosierung zu einer starken Erhöhung der Elektrodenimpedanz, kann ein Nachführen der Stimulationspegel zwar zunächst helfen. Allerdings wird mit weiterer Erhöhung der Reizströme das Risiko des Auftretens einer Fazialis-Kostimulation immer größer.

### Störungen der Hörnervfunktion

Eine mangelnde oder sogar vollkommen ausbleibende Hörnervfunktion kann u. a. durch folgende Störungen oder Ereignisse verursacht werden:

- Dysplasie oder Aplasie der N. cochlearis
- Schädel-Hirn-Trauma
- Neurochirurgische Eingriffe im Bereich der Hörbahn
- Meningitiden
- Vestibularisschwannom
- Andere retrocochleäre Degenerationen

Schädigungen des Hörnervs können sich auf sehr unterschiedliche Weise auf die Qualität der auditiven Wahrnehmung von CI-Trägern auswirken. Beispielsweise kann bei einer Dysplasie oder nach einem schweren Schädel-Hirn-Trauma mit nachfolgender Felsenbeinquerfraktur nur ein Teil des Hörnervs ausfallen. Bei den geschädigten Fasern lässt sich dann mitunter keine Hörempfindung auslösen, und die hierdurch bewirkte Störung der Tonotopie ist nur schwer oder gar nicht durch Veränderung der Kanalzuteilung zu beheben.

Auch kann die Empfindlichkeit des Hörnervs gegenüber der elektrischen Stimulation stark verringert sein, was eine Erhöhung der oberen Stimulationsgrenze mit Vergrößerung der Phasendauer der elektrischen Pulsmuster weit über den normalen Bereich erforderlich macht. Da sich durch diese Maßnahmen die Reizrate stark reduziert, kann möglicherweise kein zufriedenstellender Klang hergestellt werden.

### Langzeitdeprivation

Langzeitdeprivation beeinträchtigt oft den Erfolg einer CI-Versorgung. Eine frühzeitige Versorgung, insbesondere bei Kindern oder kurz nach dem Eintritt der Taubheit, ist entscheidend. Besonders bei Patienten mit langjähriger Taubheit, v. a. im Hochtonbereich, kann es zu Schwierigkeiten bei der Einschätzung der durch die elektrische Stimulation erzeugten Lautstärke kommen. Dies liegt an der Fremdartigkeit der Hörwahrnehmung und dem Fehlen alltäglicher Hörerfahrungen.

Zu Beginn wird empfohlen, mit den apikalen Elektroden für tiefere Frequenzen zu starten. Progressive Maps helfen, die schrittweise Gewöhnung an höhere Stimulationspegel zu unterstützen.

Bei einer kongenitalen Hörminderung mit später CI-Versorgung und wenig oder keinem Restgehör sind eine lange Akklimatisationszeit und häufige Folgeanpassungen zu erwarten. In einigen Fällen kann trotz intensiver Anpassung und Rehabilitation keine ausreichende Akzeptanz erzielt werden. Es gibt Hinweise, dass die sprachliche Kompetenz des Patienten positiv mit dem Erfolg der CI-Versorgung zusammenhängt.

### Elektrisch-akustische Stimulation

#### Indikation

Das Konzept der elektrisch-akustischen Stimulation (EAS) kombiniert elektrische Stimulation für hohe Frequenzen über ein Elektrodenarray mit akustischer Verstärkung für tiefe Frequenzen [56, 57]. Es eignet sich besonders für Patienten mit Hochtonverlust und erhaltenem Tieftonhörvermögen, bei denen Hörgeräte allein keine ausreichende Verbesserung bieten [51].

#### Anpassung bei elektrisch-akustischer Stimulation

- Spezielle CI-Prozessoren oder externe Hörer verstärken den Tieftonbereich.
- Die Übergangsfrequenz zwischen akustischer und elektrischer Stimulation wird in Abhängigkeit vom Tieftongehör individuell festgelegt.
- Mehrkanalige Systeme ermöglichen eine präzise Anpassung von Verstärkung und Kompression in der akustischen Komponente.
- Die Filterbank der CI-Komponente wird an die Übergangsfrequenz zwischen (Grenze zu noch) nutzbarem akustischem Hören und der Elektrodenlage angepasst.

##### Besonderheit.

Patienten mit nahezu intaktem Tieftonbereich und ausgeprägtem Hochtonverlust können teilweise auf die akustische Komponente verzichten. Die Übergangsfrequenz wird dann so eingestellt, dass keine störenden Überlagerungen zwischen elektrischer und akustischer Stimulation entstehen.

Bei der Erstanpassung eines EAS-Systems wird zunächst ein aktuelles Tonaudiogramm erstellt, um die Übergangsfrequenz festzulegen. Da oft noch keine Mikrootoplastik für die Akustikeinheit vorliegt, wird zuerst nur der elektrische Teil angepasst. Dies erleichtert die schrittweise Gewöhnung an den neuen Höreindruck.

Nach Fertigstellung der Otoplastik wird der akustische Teil hinzugefügt, was den Höreindruck deutlich verbessert. Patienten mit langjähriger Hochtondeprivation können anfänglich Akzeptanzprobleme haben, da die elektrische Hochtonstimulation ungewohnt ist. Diese Schwierigkeiten lassen sich durch kontinuierliches Hörtraining meist überwinden.

## Verifikation der Anpassung

Die Verifikation der CI-Einstellung ist von besonderer Bedeutung. Dadurch wird sichergestellt, dass die CI-Einstellung überhaupt ein ausreichendes Hören ermöglicht. Die Verifikation erlaubt außerdem die Identifikation geeigneter Maßnahmen zur Optimierung der CI-Einstellung. Die Verifikationsmaßnahmen entsprechen weitgehend audiometrischen Standarduntersuchungen. Sie können unabhängig von der CI-Anpassung erfolgen; für eine effiziente Anpassung und Verbesserung des CI-Ergebnisses ist jedoch eine enge Zusammenarbeit zwischen Audiometrie, Hörtherapie und CI-Anpasser sinnvoll.

### Aufblähkurve

Eine erste Überprüfung der Einstellung kann durch die Messung der Aufblähkurve (ABK) erfolgen. Dabei wird bei eingeschaltetem CI-System und regelrecht vertäubtem Gegenohr mit der aktuellen Einstellung die frequenzabhängige Hörschwelle gemessen. Diese wird als Aufblähkurve (ABK) bezeichnet. Hierzu werden Wobbeltöne oder Schmalbandrauschen verwendet. Aus Lage und Verlauf der ABK kann die Hörbarkeit der Sprachanteile abgeschätzt werden. Die ABK sollte im Frequenzbereich von 250 bis 6000 Hz zwischen 20 und 35 dB HL liegen und nicht zu große Schwankungen aufweisen, um normal gesprochene Umgangssprache ausreichend verstehen zu können. Liegt die Aufblähkurve schlechter als 40 dB HL, ist das Sprachverstehen in aller Regel eingeschränkt. Bei Kindern ist bei der Interpretation der Aufblähkurve zu berücksichtigen, dass die Reaktionsschwelle schlechter als die tatsächliche Hörschwelle sein kann.

Die Aufblähkurve ist nur eine *notwendige* Voraussetzung für die Hörbarkeit. Sie gibt keine Auskunft, wie die Signale empfunden werden, ob sie unterschieden werden können und wie die überschwellige Stimuli empfunden werden.

### Lautheitsskalierung

Die kategoriale Lautheitsskalierung (KLS) dient zur Darstellung des überschwelligen Lautheitsempfindens. Hierzu werden Stimuli mit unterschiedlichen Intensitäten im Bereich zwischen „sehr leise“ bis „zu laut“ dargeboten, und der CI-Träger ordnet diesem eine verbale Kategorie zu. Bei der Durchführung ist auf die zufällige Auswahl der Intensitäten zu achten, und Wiederholungen sind erforderlich, um die Reliabilität der Lautheitsbeurteilungen einzuschätzen.

Die genaue Durchführung der KLS ist abhängig von der Fragestellung. Sie kann bereits bei der Anpassung bei der Stimulation einzelner Elektroden durchgeführt werden, um neben der Hör- und Behaglichkeitsschwelle auch den Verlauf der Lautheitsempfindung zu erfassen.

Zur Verifikation der Anpassung wird in aller Regel im freien Schallfeld mit terzbreitem Schmalbandrauschen gemessen. Üblicherweise beschränkt man sich hier auf die 4 Oktavfrequenzen zwischen 500 und 4000 Hz (bei EAS-Versorgung auch bei 250 und 125 Hz). Es empfiehlt sich, die Lautheitsskalierung auch binaural durchzuführen (z. B. CI + Hörgerät oder CI + CI). Dadurch wird die binaurale Lautheitssummation miterfasst.

Um das Lautheitsempfinden auf beiden Seiten getrennt zu erfassen, empfiehlt sich ein binauraler Lautheitsabgleich. Wechselseitig werden links und rechts Signale präsentiert. Die Testperson muss dann nur entscheiden, welche Seite „lauter“ oder „leiser“ klingt. Daraus können dann Rückschlüsse für die Programmierung der Hörsysteme gezogen werden.

**Abb. 5:**
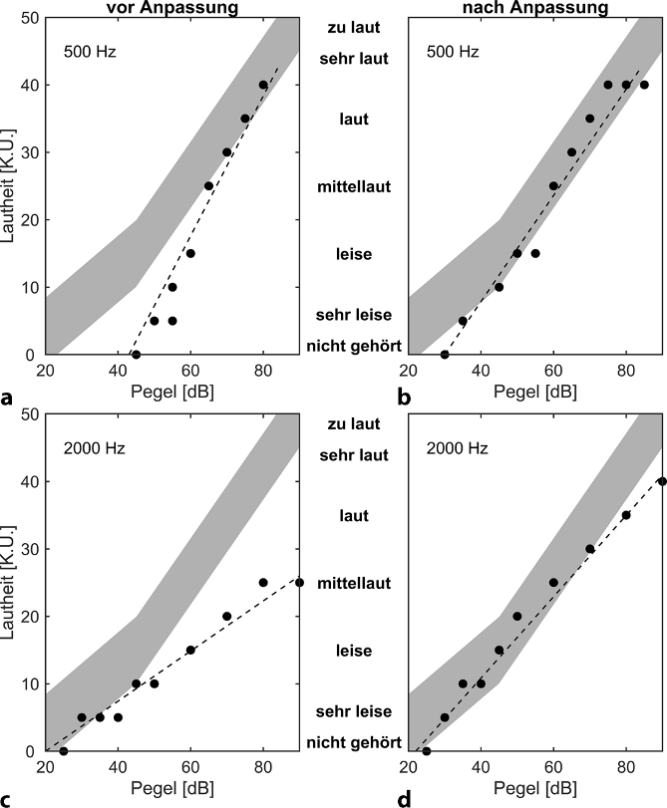
Ergebnisse für Lautheitsskalierungen für 2 Frequenzen. **a, b** Die Lautheitsbeurteilungen bei 500 Hz (*schwarze Punkte*) **a** im Vergleich zu Normalhörenden (*grauer Bereich*) bis 70 dB zu niedrig liegend, erst bei höheren Pegeln Erreichen der Werte von Normalhörenden. **b** Durch eine Korrektur der unteren Schwellen (T-Werte) Erreichen einer Normalisierung. **c, d** Die Lautheitsbewertungen bei 2000 Hz (*schwarze Punkte*) oberhalb von 40 dB **c** deutlich unter dem Normalbereich liegend. **d** Durch eine Korrektur der oberen Begrenzungen bei den zugehörigen Elektroden Erreichen einer Normalisierung auch hier, damit nun auch Lautheitsbeurteilungen von „laut“ möglich

Im Bereich der schallverstärkenden Hörsysteme wird die Messung des Lautheitsempfindens mit dem Ziel durchgeführt, eine Lautheitsnormalisierung zu erreichen, d. h. die Lautheitsfunktion soll Normalhörigen möglichst nahekommen. Für das CI-Hören ist dieses Ziel oft nicht erreichbar. Insbesondere ist die Lautheitsbeurteilung am Beginn der Basisanpassung häufig wegen langjähriger Deprivation für die CI-Träger schwierig. Mit zunehmender CI-Erfahrung liefert die Lautheitsskalierung jedoch aussagekräftigere Ergebnisse. Besonders auffällige Ergebnisse der KLS können genutzt werden, um untere oder obere Stimulationsgrenzen einzelner Elektroden zu verändern oder bei Bedarf auch den Verlauf der elektrischen Kennlinie über dem Eingangspegel. In Abb. 5 sind die Ergebnisse der KLS vor und nach Anpassung dargestellt.

### Sprachverstehen

Die Messung des Sprachverstehens ist ein wichtiger Bestandteil der Verifikation einer CI-Anpassung. Ein wichtiges Werkzeug ist hier der Freiburger Sprachverständlichkeitstest [33]. Üblicherweise wird die Sprachaudiometrie über Lautsprecher im freien Schallfeld durchgeführt. Wenn sie monaural bei vertäubtem Gegenohr durchgeführt wird, gibt sie nicht nur Auskunft über die Hörleistungen, sondern erlaubt auch Rückschlüsse für die CI-Einstellung.

#### Sprachverstehen in der Nähe der Hörschwelle

Die mehrsilbigen Zahlwörter dienen zur Erfassung der basalen Hörleistungen. In erster Linie können sie als Maß für die Hörbarkeit verwendet werden. Sie finden hauptsächlich in den ersten Tagen und Wochen der CI-Anpassung Einsatz. Bei weit überschwelligen Pegeln etwa 65 dB SPL sollte bald nahezu 100 % Verständlichkeit erreicht werden. Ist dies der Fall, kann das Zahlenverstehen bei niedrigeren Pegeln gemessen werden. Der Pegel, bei dem 50 % der Zahlwörter verstanden wird, wird als Sprachverständlichkeitsschwelle (SVS) bezeichnet. Die SVS liegt im Regelfall etwa 20 dB über der den mittleren Werten der ABK. Sie gibt somit Aufschluss über die Einstellung der unteren Begrenzungen der CI-Einstellung. Im optimalen Fall liegt die SVS mit CI bei 35–45 dB oder noch niedriger. Die SVS lässt sich auch mit dem Oldenburger Satztest (OlSa) bestimmen. Die einfachen Sätze werden ähnlich gut verstanden wie Zahlwörter. Der OlSa kann in offener Form oder in geschlossener Form durchgeführt werden. Bei der geschlossenen Variante werden der Testperson alle Wörter zur Auswahl visuell dargeboten. Die Testperson muss dann aus den dargestellten Wörtern auswählen. Wenn die Testdurchführung über einen Touchscreenmonitor und eine automatisierte Steuerung geschieht, können die CI-Träger die SVS-Messung nahezu eigenständig durchführen. Die SVS für den OlSa sollte ebenfalls im Bereich von 35–45 dB SPL liegen.

Die Messung des Verstehens für Zahlwörter und Oldenburger Sätze in der geschlossenen Variante ist darüber hinaus noch geeignet, um bei Menschen mit sehr geringem Sprachverstehen die Hörleistungen abzuschätzen. Hierzu zählen z. B. Menschen mit prä- oder perilingualem Einsetzen der Taubheit, Langzeitdeprivation, anatomisch-physiologischen Besonderheiten oder Fehllagen des CI-Elektrodenarrays.

#### Einsilberverstehen

Der Freiburger Einsilbertest (FBE) ist eine wichtige Größe in der Beschreibung der Ergebnisqualität. Das prozentuale Einsilberverstehen bei 65 dB SPL ist im CI-Weißbuch der DGHNO-KHC als postoperativ regelmäßig zu erhebende Größe aufgeführt [47]. Um den statistischen Mindeststandards zu entsprechen, sollten mindestens 2 Listen à 20 Wörter verwendet werden. Neuere Computeraudiometer erlauben die Permutierung der Testwörter. Dadurch wird unerwünschtes „Auswendiglernen“ vermieden.

**Abb. 6:**
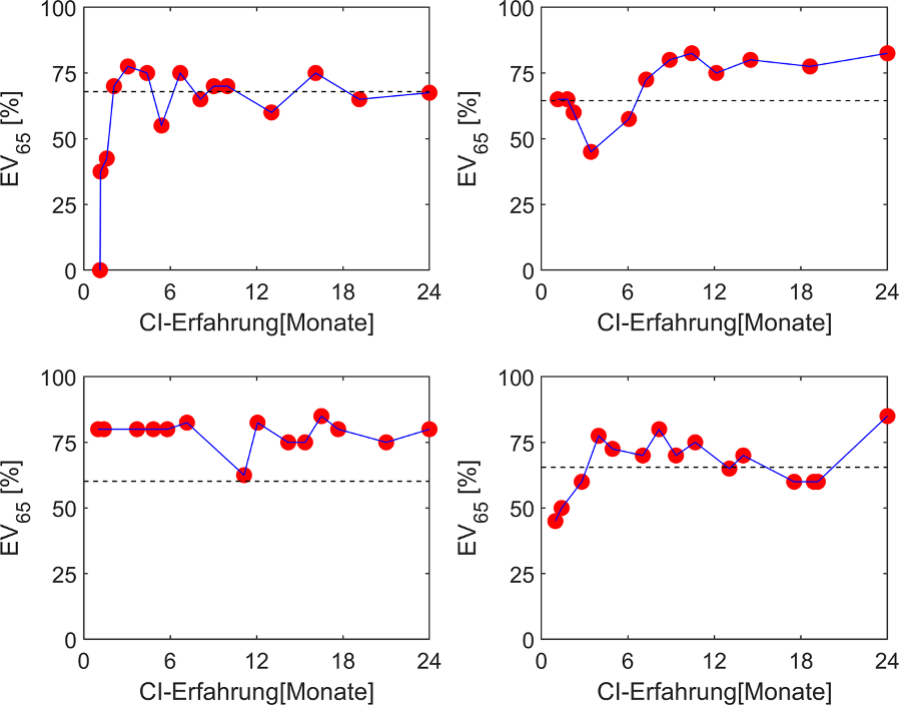
Einsilberverstehen bei 65 dB SPL mit Cochleaimplantat(CI-)System für 4 erwachsene, postlingual ertaubte CI-Träger im Verlauf der ersten 2 Jahre. *Gestrichelte Linie *präoperativ bestimmte Prädiktion. Ziel: nach 3–6 Monaten Erreichen eines stabilen Sprachverstehens in der Nähe der Prädiktion

Darüber hinaus kann der Einsilbertest auch zur Verifikation der CI-Anpassung verwendet werden. Arbeiten unterschiedlicher Arbeitsgruppen haben gezeigt, dass für CI-Patienten mit präoperativ noch messbarem Restsprachverstehen über Kopfhörer eine Prognose des mit CI-System erreichbaren Sprachverstehens möglich ist [17, 20, 29, 34, 59]. Aus 3 präoperativ i. d. R. bekannten Größen lässt sich ein Score berechnen, dem das Einsilberverstehen mit CI nach 6 Monaten bei erfolgreicher CI-Anpassung nahekommt. Hierzu wird lediglich das Alter des Patienten bei CI-Versorgung, das im Sprachaudiogramm maximal erreichbare Einsilberverstehen (maximales Einsilberverstehen) und das mit Hörgerät monaural bei 65 dB SPL erreichte Einsilberverstehen benötigt. Daraus ist bereits bei der Erstanpassung ein individueller Erfolgshorizont für das Einsilberverstehen definiert. Eine Realisierung dieses Scores ist auf der Homepage des Greifswalder Hörzentrums (https://www.medizin.uni-greifswald.de/hno/hoerzentrum-nord-ost/hoppe-score/) verfügbar. Dieses Modell wurde empirisch auf der Datenbasis der Ergebnisse von nahezu 1000 erwachsenen CI-Patienten ermittelt. In Abb. 6 ist für 4 erwachsene CI-Träger der Verlauf des Einsilberverstehens über die Zeit dargestellt. Zusätzlich ist die Prädiktion als gestrichelte Linie dargestellt. Aktuell wird in verschiedenen Studien die Anwendung anspruchsvollerer Datenanalysen mit maschinellem Lernen unter Einbeziehung größerer Datensätze versucht, die Prädiktion der erreichbaren Hörleistung mit CI zu verbessern.

#### Sprachverstehen im Störgeräusch

Ein Ziel der Anpassung ist nicht nur eine Optimierung des Sprachverstehens in Ruhe, sondern auch das Verstehen im Störgeräusch zu verbessern. Daher sollte dies ebenfalls überprüft werden. Die Verwendung des FBE im Störgeräusch ist zwar möglich, jedoch sind Satztests geeigneter, weil sie realistischere Situationen darstellen. Die weitesten verbreiteten Tests sind der Oldenburger Satztest (OlSa), der Göttinger Satztest (GöSa) und der HSM-Satztest (Hochmair-Schulz-Moser-Test). Diese können prinzipiell auch in Ruhe verwendet werden. Die Verständlichkeit ist deutlich besser als für einsilbige Prüfwörter und ist bei Umgangssprachpegeln nahe 100 %.

Die Messung im Störgeräusch kann mit festem Sprach- und Störgeräuschpegel bei definiertem Signal-Rausch-Verhältnis (SNR) erfolgen oder bei variierendem SNR mit Bestimmung der SVS.

Bei festem SNR orientiert man sich an den im Alltag vorkommenden Hörsituationen. Häufige Werte im Alltag reichen von −5 bis +10 dB.

Die Messung der SVS erfolgt am besten über eine adaptive, computergesteuerte Prozedur, bei der z. B. der Sprachpegel fest auf 65 dB gesetzt wird und der Störgeräuschpegel so lange variiert wird, bis eine Verständlichkeit von 50 % erreicht wird. Das zugehörige SNR wird als SRT bezeichnet. Der Vorteil solcher adaptiven Messungen liegt darin, dass im Bereich der SVS Veränderungen des SNR den größten Einfluss auf die Verständlichkeit haben. Dadurch können Veränderungen eher erfasst werden.

Für das Sprachverstehen im Störgeräusch spielt die binaurale Interaktion eine besondere Rolle. Um diese gezielt darzustellen, muss die räumliche Anordnung der Lautsprecher entsprechend gewählt werden. Sind die Lautsprecher für Sprache und Störschall identisch und vor der Testperson (S_0_N_0_) befindlich, so wird im Wesentlichen die binaurale Summation erfasst. Andere Anordnungen wie S_0_N_90_ oder S_0_N_-90_ (Sprache von vorn, Rauschen von rechts bzw. links) werden verwendet, um Effekte wie Kopfschatten oder binaurale Störgeräuschunterdrückung („binaural masking level difference“) darzustellen. Durch den Vergleich zwischen monauraler Hörsystemversorgung und binauraler Hörsystemversorgung lassen sich mit den geeigneten Messungen im Störgeräusch die Verbesserungen durch die binaurale Versorgung objektivieren.

### Richtungshören

Die Überprüfung des Richtungshörens in der Horizontalebene wird durchgeführt, um die interaurale Interaktion zu überprüfen. Bei CI-Trägern spielen insbesondere interaurale Pegelunterschiede eine wichtige Rolle. Insbesondere bei der einseitigen Taubheit ist die Verbesserung des Richtungshörens ein wichtiges Ziel und sollte daher getestet werden.

### Validierung der CI-Anpassung mittels Fragebogen

Unter dem Begriff „Verifikation der CI-Anpassung“ versteht man die Bestätigung, dass das CI-System so funktioniert, wie es soll. Der individuelle Nutzen einer CI-Versorgung ist letztlich durch die audiometrischen Messungen nicht ausreichend zu bestimmen. Unter „Validierung der CI-Versorgung“ versteht man die Überprüfung, ob der Nutzer selbst auch im Alltag einen Nutzen empfindet und ob diese seine Lebensqualität verbessert. Um die Verbesserung der Lebensqualität darzustellen, werden Fragebogen eingesetzt. Es existieren zahlreiche Fragebogeninventare, die jeweils spezifische Anwendungsbereiche und Zielgruppen haben. Exemplarisch werden hier 2 Inventare vorgestellt, die derzeit die größte Anwendung im Bereich der Hörsystemvalidierung finden.

#### Nijmegen Cochlear Implant Questionnaire

Im Bereich der CI-Versorgung ist der Nijmegen Cochlear Implant Questionnaire (NCIQ) am weitesten verbreitet [30]. Er erfasst anhand von 60 Fragen die Bereiche

- elementare Schallwahrnehmung,
- Sprach- und Musikwahrnehmung,
- Kontrolle der eigenen Stimme,
- psychosoziale Folgen („self esteem“),
- Aktivitätsverhalten,
- soziale Kontakte.

Somit wird nicht nur die Verbesserung der Hörqualität, sondern auch der Einfluss der Versorgung auf verschiedene Bereiche des Alltags erfasst. Die Subgruppen 1–3 beschreiben die physische Domäne, die 4. Gruppe repräsentiert die psychische Domäne und die Fragen der 5. und 6. Gruppe die soziale Domäne. Zusätzlich lässt sich ein Gesamtscore bestimmen, der integral die Verbesserung erfasst. Der NCIQ wurde von einer Berliner Arbeitsgruppe in die deutsche Sprache übertragen [31]. Er ist Teil des CI-Registers und soll präoperativ und regelmäßig postoperativ erhoben werden. Für die Einstellung des CI-Systems lassen sich nur sehr begrenzt Rückschlüsse aus dem NCIQ ziehen. Das Ergebnis des NCIQ kann jedoch verwendet werden, um systematisch die individuellen Hörprobleme zu ermitteln, die dann z. B. zur Aktivierung geeigneter Signalvorverarbeitungsverfahren führen können.

#### Abbreviated Profile of Hearing Aid Benefit

Der ursprünglich für die Hörgeräteversorgung entwickelte Fragebogen Abbreviated Profile of Hearing Aid Benefit (APHAB) [12] kann auch im Bereich der CI-Versorgung eingesetzt werden [46]. Dies wird gerechtfertigt durch die Indikationserweiterungen im CI-Bereich. Dadurch werden CI-Patienten mehr und mehr audiologisch vergleichbar mit Schwerhörigen, die ein konventionelles Hörgerät nutzen. Dieser Fragebogen besteht aus 24 Fragen, von denen jeweils 6 zu einer Subskala gehören. Die 4 Subskalen des APHAB sind:

- Sprachverstehen in ruhiger Umgebung (Ease of Communication, EC)
- Sprachverstehen in Störgeräuschen (Background Noise, BN)
- Sprachverstehen in halligen Umgebungen (Reverberation, RV)
- Unangenehme Hörempfindung von ‚lauten‘ Geräuschen (Aversiveness, AV)

Der APHAB erfasst somit vorwiegend Aspekte des Sprachverstehens. Die 4. Subskala erlaubt jedoch auch eine Einschätzung der Beeinträchtigung durch starken Störschall und laute Nebengeräuschen wie Reifenquietschen oder Martinshörner. Dies wiederum erlaubt Rückschlüsse für die Programmierung des CI-Systems, insbesondere der oberen Stimulationsbegrenzungen.

Zusammenfassend ist die Verifikation der Anpassung mit audiometrischen Methoden unerlässlich für eine erfolgreiche CI-Anpassung. Dadurch werden einzelne Aspekte der CI-Anpassung kontrolliert, und die Anpassung kann gezielt modifiziert und optimiert werden. Die Maßnahmen zur Verifikation sind daher von großer Bedeutung in der CI-Versorgung und gehen deutlich über die Darstellung der aktuellen Hörfähigkeiten mittels Sprachverständlichkeitstests hinaus. Für die Validierung der CI-Versorgung ist der Einsatz von geeigneten Fragebögen unerlässlich.

## Fazit für die Praxis

- Die Anpassung von Cochleaimplantat(CI)-Systemen erfordert ein hohes Maß an medizinischem und technischem Fachwissen, das weit über die Anpassung von schallverstärkenden Hörsystemen hinausgeht.
- Dabei spielen sowohl Individualisierung als auch Standardisierung eine wichtige Rolle.
- Die Anpassung muss individuell auf die medizinisch-audiologischen Voraussetzungen und die Ansprüche des Patienten abgestimmt werden.
- Gleichzeitig dienen standardisierte Verfahren und Techniken als Grundlage für das sichere Erreichen des Ziels einer bestmöglichen CI-Anpassung.
- Der Fokus liegt zu Beginn auf der Akzeptanz des CI-Hörens, gefolgt von der Optimierung des Sprachverstehens in Ruhe und bei Störgeräuschen.
- Profunde Kenntnisse der Hörphysiologie sind erforderlich, um Schäden durch falsche oder zu starke Stimulationsströme – besonders bei Kindern – zu vermeiden.
- Hierfür ist die Festlegung der erforderlichen Qualifikation des den Patienten betreuenden Personals (z. B. CI-spezialisierter Audiologe) notwendig.
- Die erforderlichen Qualifikationen sind daher im Detail in der CI-Leitlinie der Arbeitsgemeinschaft der Wissenschaftlichen Medizinischen Fachgesellschaften e. V. (AWMF) und dem Weißbuch CI-Versorgung der Deutschen Gesellschaft für Hals-Nasen-Ohren-Heilkunde, Kopf- und Hals-Chirurgie e. V. (DGHNO-KHC) definiert.
